# Pd-Enhanced Carbon-Encapsulated Co Nanoparticles for Efficient Reverse Water–Gas Shift under Magnetic Induction Heating

**DOI:** 10.1021/acscatal.5c01232

**Published:** 2025-05-20

**Authors:** Adrián García-Zaragoza, José Luis del Río-Rodríguez, Christian Cerezo-Navarrete, Silvia Gutiérrez-Tarriño, M. Asunción Molina, Lucy Costley-Wood, Jaime Mazarío, Bruno Chaudret, Luis M. Martínez-Prieto, Andrew M. Beale, Pascual Oña-Burgos

**Affiliations:** a ITQ, Instituto de Tecnología Química, Universitat Politècnica de València (UPV), Av. de los Naranjos S/N, Valencia 46022, Spain; b Department of Chemistry, 4919 University College London, 20 Gordon Street, London WC1H 0AJ, U.K.; c Research Complex at Harwell, Rutherford Appleton Laboratories, Harwell Science and Innovation Campus, Harwell, Didcot OX11 0FA, U.K.; d LPCNO, Laboratoire de Physique et Chimie des Nano-Objets, 137668 Université de Toulouse, CNRS, INSA, UPS, Toulouse 31077, France; e IIQ, Instituto de Investigaciones Químicas (CSIC-Universidad de Sevilla), Avda. Americo Vespucio 49, Seville 41092, Spain

**Keywords:** reverse water−gas shift, CO_2_ reduction, magnetic induction heating, magnetic catalysts, in situ X-ray absorption

## Abstract

Reducing CO_2_ to CO via the reverse water–gas shift (RWGS) reaction is a promising strategy for carbon capture and utilization (CCU). In this study, tailored magnetic catalysts were designed through the pyrolysis of a Co-based MOF to form well-defined nanoparticles. As a result, carbon-encapsulated cobalt nanoparticles (**Co@C**) and palladium-doped cobalt nanoparticles (**CoPd/Co@C**) were synthesized and thoroughly characterized using a variety of techniques, including *in situ* X-ray absorption and diffraction experiments. These carbon-based catalysts were simultaneously used as heating agents and catalysts for the magnetically induced RWGS reaction, exhibiting remarkable activity and selectivity for syngas production. CO_2_ conversions of 61.1% and 71.1% were obtained for **Co@C** and **CoPd/Co@C** (63 mT, 2 kW, 320 kHz), respectively. Using magnetic induction heating (MIH), these catalysts operate at lower local temperatures and with greater energy efficiency than conventional thermal heating, while achieving superior CO production efficiency. Notably, **CoPd/Co@C** achieved highly satisfactory CO production efficiency (478.5 mL_CO_/kW·h), demonstrating a significant improvement compared to the analogous process utilizing magnetically induced heating. Furthermore, **CoPd/Co@C** exhibited unwavering stability, maintaining its performance for more than 200 h without significant degradation or need for reactivation. This study highlights the potential of MIH for industrial applications in CO_2_ reduction, offering a more renewable and economically viable alternative to traditional methods.

## Graphical abstract

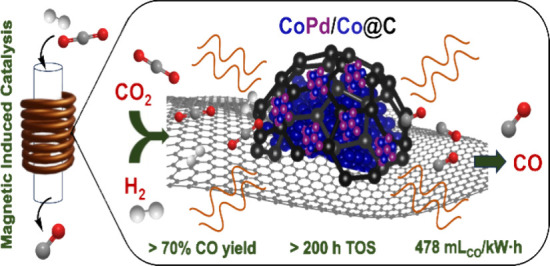

## Introduction

CO_2_ is one of the most significant contributors to the greenhouse effect (GHE), making it necessary to reduce these emissions to mitigate the impact of climate change. Therefore, carbon capture and utilization (CCU) represents a significant ongoing challenge for the scientific community.^1–3^ In this sense, the hydrogenation of CO_2_ into higher value-added products is of great interest. One of the most studied reactions in CO_2_ hydrogenation is the Reverse Water–Gas Shift (RWGS) reaction, where CO_2_ is reduced to form CO and H_2_O.^4–7^ This reaction has gained significant attention as CO is one of the most widely used C1 building blocks, serving as a crucial raw material for producing clean liquid fuels and value-added chemicals through Fischer–Tropsch synthesis (FTS).^8–10^

The RWGS reaction is favored at high temperatures due to its slightly endothermic nature and the high energy demand required for activating the CO_2_ molecule.^11^ Temperatures exceeding 600 °C are typically required to obtain good CO yields during the reaction. At lower temperatures, CO_2_ can be reduced to CH_4_ through the methanation process, also known as the Sabatier reaction.^12^ Unfortunately, during RWGS, high temperatures compromise the integrity of the catalyst (i.e., sintering) and the reactor (i.e., mechanical corrosion).^13,14^ For all these reasons, it is necessary to explore novel technologies that are energetically more efficient, capable of performing the RWGS reaction at milder temperatures, and consequently, with lower costs and less environmental impact.

Magnetic induction heating (MIH) is an emerging technology in the field of catalysis as an attractive, more efficient alternative to conventional heating.^15,16^ MIH is based on the ability of magnetic materials to release heat through hysteresis losses in the presence of an alternating magnetic field (AMF). The use of catalysts based on magnetic nanoparticles (MagNPs) to transform electromagnetic energy into heat offers several advantages, making MIH suitable for intermittent energy sources.^17,18^ These benefits include: (i) rapid heating, (ii) direct heat transfer from the heating agent to the catalyst, and (iii) the electrification of the process, which reduces energy costs. In addition, it has recently been proposed that MIH can promote alternative reaction mechanisms due to changes in the spin state of the active catalyst, as well as provide an alternative heating source.^19^ All this makes MIH an attractive technology for performing catalytic reactions using MagNPs-based catalysts in both solution and gas phases.^20–23^ In particular, MIH has proven that excellent results can be achieved in catalytic reactions operating under apparent mild reaction conditions where normally higher temperatures and pressures are required.^24,25^ This is because the catalyst surface is at a significantly higher temperature (T_surf_) than the recorded local temperature of the entire system (T_local_).^26^ However, this localized heating can also lead to agglomeration and sintering of the magnetic catalysts due to the high temperatures reached on the surface, causing structural and chemical degradation and reducing the catalyst lifetime.^27,28^ To overcome this limitation, some of us have reported the encapsulation of MagNPs within a carbon layer that helps to inhibit partial oxidation and sintering at high temperatures, thus enhancing their stability.^29,30^ In this sense, there has recently been growing interest in using Metal–Organic Frameworks (MOFs) as a template to synthesize heterogeneous catalysts based on metal nanoparticles (NPs) encapsulated within a carbonaceous support.^31–33^ The metallic functionalities, often in the form of nanoparticles, are expected to originate from the initial metal nodes, whereas the organic linker will end up as a support or matrix to encapsulate these metallic components. These catalysts have demonstrated outstanding results in terms of activity and catalytic stability, yet their application in magnetic induction heating remains unexplored.

There are many studies on the performance of catalysts based on supported nanoparticles (Cu, Fe, Ni, etc.) for the RWGS reaction using conventional thermal heating. One of the most active catalysts reported to date is that studied by Rossi et al., based on nickel carbide nanoparticles (formed *in situ*) supported on silica, achieving conversions close to 80% while being completely selective to CO.^34,35^ However, substantial energy consumption (due to temperatures of up to 800 °C) was required to achieve this catalytic performance. The use of bimetallic catalysts, such as Co–Fe/Al_2_O_3_ ^36^ or Pd–Co/SBA-15,^37^ has also been studied, but the conversions do not exceed 55% and require high temperatures (i.e., 700 °C). With the aim of reducing this high energy input, it has recently been reported that applying an external electric field can promote the activity of catalysts in the RWGS reaction. Specifically, Yamaoka et al. reported that by applying an electric field of 3–7 mA, they were able to achieve conversions close to 20% at temperatures as low as 150 °C, observing a reduction in the apparent activation energy of the reaction from 61.4 to 5.9 KJ·mol^–1^, using an Fe-based catalyst.^38^ In a similar vein, magnetic induction heating has also been used in the hydrogenation of CO_2_, primarily for the production of CH_4_. A particularly relevant example is one reported previously by some of the authors. In the study, FeCo nanoparticles encapsulated in carbon (FeCo@C) were used as the heating agent and Ni nanoparticles as the catalytically active species (FeCo@C/Ni). A conversion of 96% was achieved with almost complete selectivity to CH_4_.^29^ Regarding the magnetically induced RWGS reaction, the use of core–shell type CoNi MagNPs (Co@Ni@C) has shown excellent results in CO production by using an AC magnetic field with a maximum power of 8 kW (74.6% conversion with complete selectivity to CO).^39^ However, the stability of the material was tested for less than 10 h. Hence, these results are limited as far as the industrial-level application is concerned.

Herein, we present an efficient approach for synthesizing a novel magnetic catalyst based on nondoped Co nanoparticles encapsulated in carbon (**Co@C**), and one doped with palladium (**CoPd/Co@C**), leveraging the widely known ability of palladium to facilitate the activation of hydrogen molecules.^40^ To achieve this, we modified a previously reported 2D-CoMOF with a palladium salt and then generated the carbon-based metal catalyst through a pyrolysis step. This synthetic approach allows the creation of well-defined nanoparticles encapsulated in carbon, which protects them from sintering at high temperatures. The synthesized magnetic catalysts have been fully characterized by advanced characterization techniques, such as STEM, HRTEM, XPS, PXRD, XAS, and magnetic (VSM) and calorimetric (SAR) measurements. **Co@C** and **CoPd/Co@C** have proven effective as heating and catalytic agents for the magnetically induced reduction of CO_2_ into CO, showing exceptional catalytic results. Furthermore, the inclusion of palladium has improved the catalyst’s activity and stability, with **CoPd/Co@C** remaining stable for over 200 h without significant loss of activity and any need for reactivation.

## Results and Discussion

### Synthesis and Characterization

**Co@C** and **CoPd/Co@C** catalysts were synthesized using a two-step procedure (Scheme 1). In the first stage, a well-defined 2D-CoMOF precursor supported on carbon (2D-CoMOF/C) was obtained following a procedure developed in our group and reported elsewhere,^41^ but including carbon Vulcan XC-72 in the synthesis. To synthesize the precursor of the bimetallic catalyst, Pd/2D-CoMOF/C, Na_2_PdCl_4_ salt was also added in this synthetic step (more information in materials and methods). In a second stage, aimed to protect the nanoparticles from sintering at high temperatures, the resulting precursors were pyrolyzed at 800 °C (heating rate: 25 °C/min, N_2_ flow: 20 mL/min) to produce the final catalyst, where nanoparticles were encapsulated by a carbon-shell (**Co@C** and **CoPd/Co@C**). Due to the high temperatures reached during magnetically induced catalysis, it is important to have a well-defined carbon shell around the metallic nanoparticles, protecting them from sintering (*vide infra*). The metal content of the nanoparticles was determined by X-ray fluorescence (XRF). **Co@C** has a cobalt content of 11.1 wt % Co. In comparison, **CoPd/Co@C** shows a similar cobalt percentage, along with a small amount of palladium (10.3 wt % Co, 2.2 wt % Pd), resulting in CoPd nanoparticles (NPs) with an experimental metal composition of Co_0.90_Pd_0.10_.

**Scheme 1:**
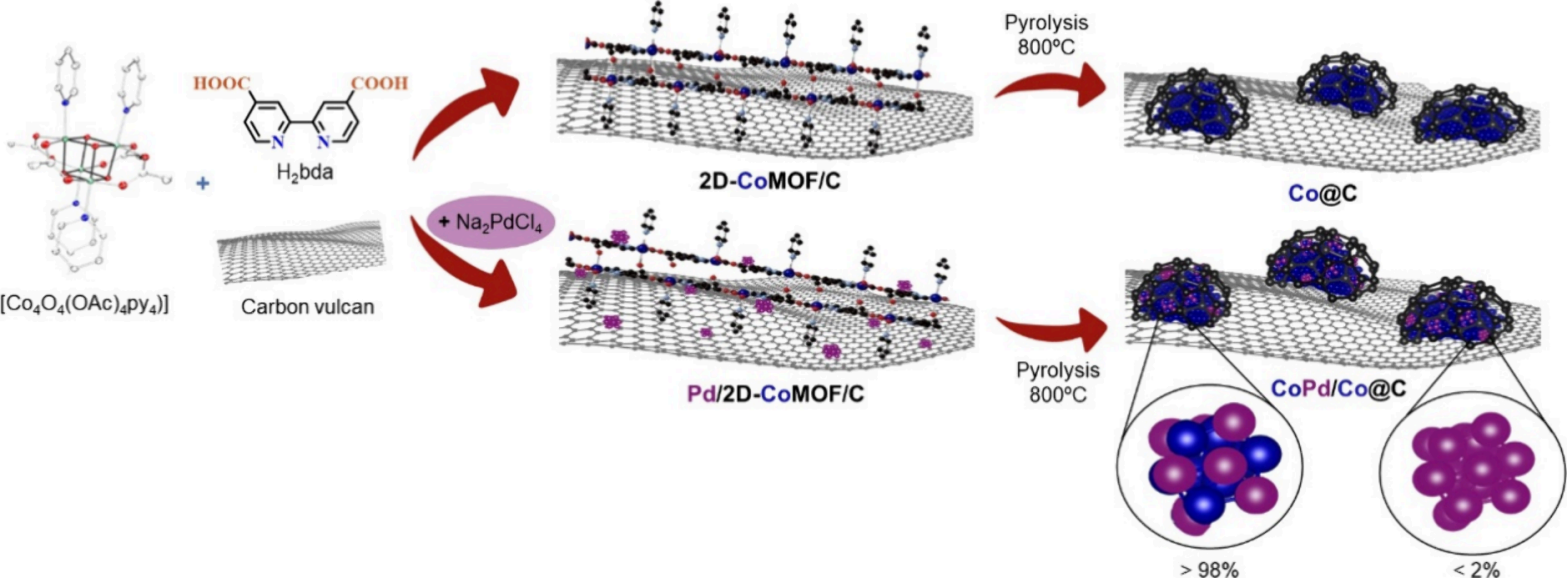
Schematic Synthesis of the **Co@C** and **CoPd/Co@C** Catalysts^a^

a Cobalt is represented in blue, and palladium is represented in purple. The contribution of CoPd alloy (>98%) and Pd nanoparticles (<2%) on the Co NPS surface is indicated in the scheme.

The incorporation of a second metal was presumed to modify the stability of the MOF, potentially resulting in a different decomposition behavior during pyrolysis and influencing the performance of the nanoparticles, both as heating agents and as catalysts. *Ex situ* PXRD patterns of the mono- and bimetallic systems before pyrolysis, 2D-CoMOF/C and Pd/2D-CoMOF/C, show only reflections characteristic of crystalline MOFs (see Figure S3.1), and the lack of metallic Co reflections confirms the well-formed Co-MOF structure in both samples. However, in the Pd-doped sample, additional reflections corresponding to face-centered cubic (*fcc*) Pd^0^ confirm the formation of crystalline Pd nanoparticles during the initial synthesis, with an approximate diameter of 15 nm (SI section 3). These nanoparticles are located on the surface of the MOF, as confirmed by field emission scanning electron microscopy (FESEM) images (SI section 4). *Ex situ* PXRD of the samples after pyrolysis contains a broad reflection of partially crystalline carbon at 2θ: 25°,^41^ due to the MOF decomposition (Figure 1b). Intense reflections of *fcc*-Co^0^ are present, and in the case of the bimetallic CoPd sample, reflections derived from alloy-CoPd can also be observed. A small quantity of Pd^0^, initially present in the synthesized sample, also remains. The sequence of phase transformations for the bimetallic sample was followed by an *in situ* combined X-ray diffraction and absorption experiment during the MOF pyrolysis (Figure 1a,c). EXAFS also confirmed the formation of a CoPd alloy by the appearance of a destructive Co–Pd path at 5.8Å^–1^ at temperatures above 400 °C (Figure 1c,d). The composition of the alloyed phase is Co_0.33_Pd_0.67_, calculated along with crystallite size by full-profile refinements (SI section 3, Figure S3.3). This composition accounts for only the CoPd alloy phase and hence is different from the Co:Pd ratio calculated for the total sample by XRF (*vide supra*). The Co^0^, the majority phase in both **Co@C** and **CoPd/Co@C**, had crystallite sizes of 15 and 17 nm, respectively, with the CoPd alloy phase having a crystallite size of 7 nm.

**Figure 1:**
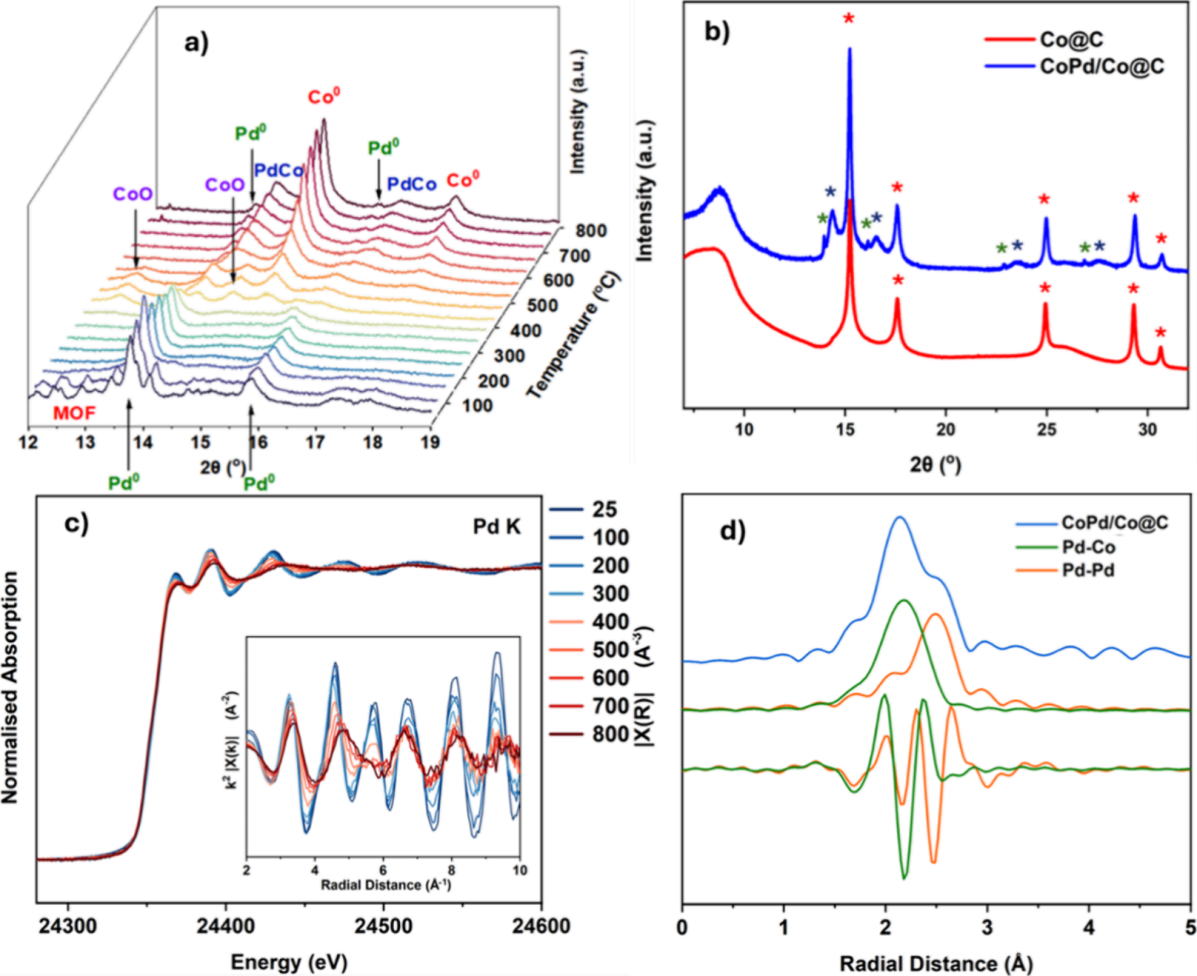
Powder XRD during (a) in situ pyrolysis of Pd/2D-CoMOF/C, collected at 23 keV each 100 °C (various crystalline phases are labeled), and (b) from ex situ measurement of the pyrolyzed **Co@C** and **CoPd/Co@C**, also at 23 keV. The Pd^0^ phase is identified in green, the CoPd is in blue and the Co^0^ is in red. (c) Normalized absorption data and k^2^ weighted χ­(k) data during the same in situ pyrolysis of Pd/2D-CoMOF/C to **CoPd/Co@C** at the Pd K edge, showing a transition from Pd^0^ to CoPd by the addition of a destructive path at 5.8 Å^–1^ as the temperature surpasses 500 °C, and (d) ex situ k^2^-weighted χ­(k) data and Fourier transform magnitude of the pyrolyzed sample at the Pd K-edge, showing separate contributions from the Pd–Co and Pd–Pd path as generated by FEFF calculations.

To withstand the high temperatures of magnetically induced catalysis, the formation of a carbon shell to protect the metal nanoparticles from sintering was a key reason for selecting the approach presented herein. In that sense, scanning transmission electron microscopy (STEM) confirms this carbon coating. The micrographs confirm the formation of well-dispersed, spherical Co nanoparticles in **Co@C** with an average size of 8.8 ± 4.9 nm (Figure 2a). In the case of the bimetallic catalyst (**CoPd/Co@C**), adding palladium increases the nanoparticle size to an average of 12.7 ± 6.5 nm (Figure 2e). These sizes are slightly smaller than those calculated by PXRD. However, the statistical error is higher, and the larger size of cobalt in the bimetallic sample (**CoPd/Co@C**) is consistent between the two techniques. The thickness of the carbon layer is ca. 3 nm, which is similar for both catalysts (Figure 2b,f). Additionally, the chemical composition was studied using STEM-HAADF coupled with EDX analysis. **Co@C** shows a good distribution of cobalt in the carbonaceous material (Figure 2d). For **CoPd/Co@C**, the EDX analysis and line scan profile show that both metals are present (Figure 2h, Figure S13.1).

**Figure 2:**
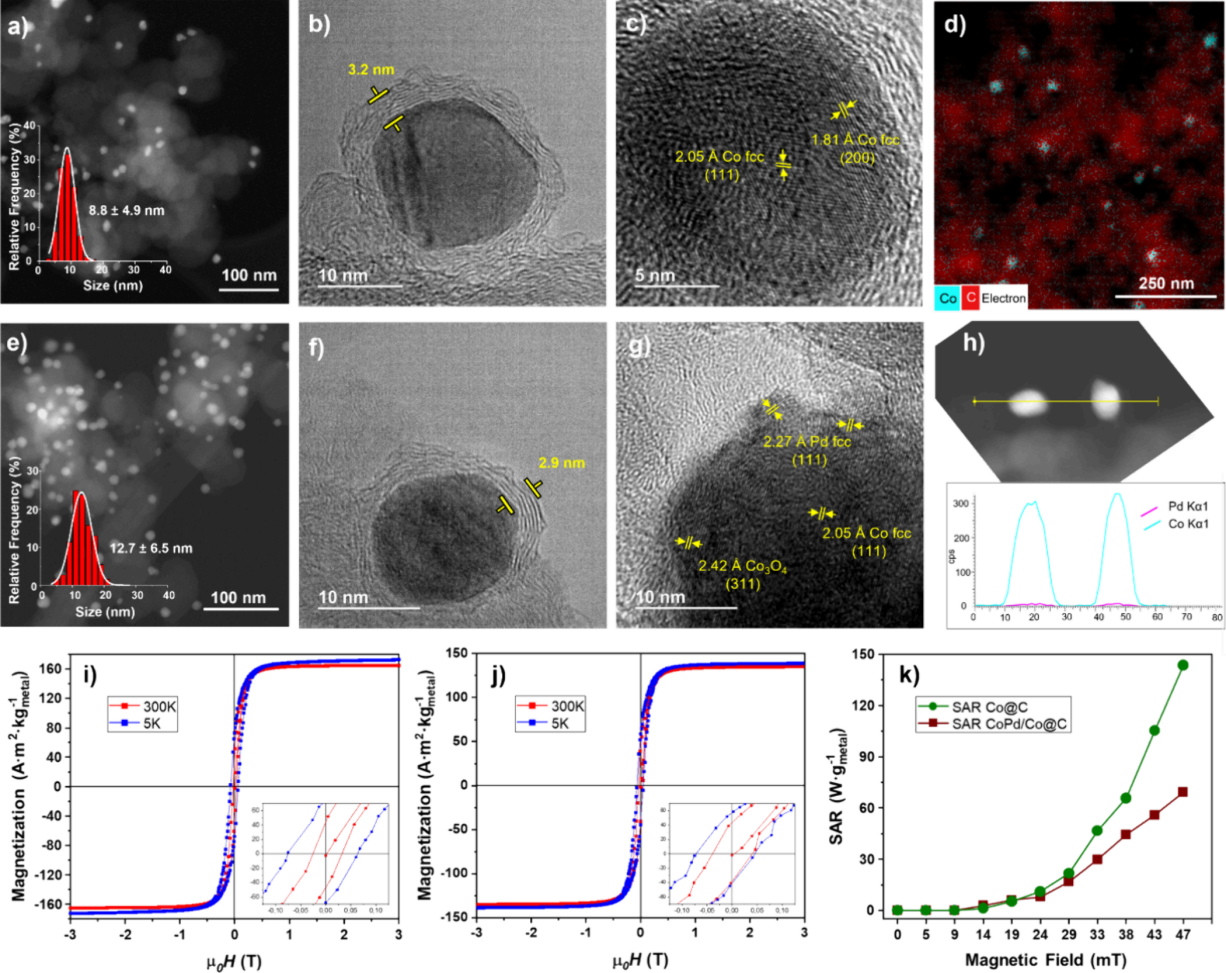
STEM image and size histogram of (a) **Co@C** and (e) **CoPd/Co@C**. HRTEM micrographs of (b, c) **Co@C** and (f,g) **CoPd/Co@C**, where lattice spacings and thickness of the carbon shell are highlighted in yellow. STEM-HAADF coupled with EDX analysis of (d) **Co@C**, where Co is marked in blue and C in red. (h) STEM-HAADF image and EDX line scan profile of **CoPd/Co@C**. (i) VSM hysteresis loops measured by VSM of **Co@C**. (j) VSM hysteresis loops measured by VSM of **CoPd/Co@C**. (k) SAR measurements of **Co@C** (garnet) and **CoPd/Co@C** (green).

The distribution of the different crystalline phases in isolated nanoparticles of the pyrolyzed catalysts was studied through high-resolution TEM (HRTEM) close-ups of a single nanocrystal (Figure 2c, g). The crystalline phases previously identified by XRD can be associated with the lattice fringes observed in HRTEM. In **Co@C**, lattice fringes with interplanar spacings of 2.05 Å and 1.81 Å were identified, which could correspond to the (111) and (200) planes of the *fcc*-Co phase (Figure 2c) (JCPDS: 00–015–0806). In contrast, two distinct interplanar distances are observed in the HRTEM image of **CoPd/Co@C**: one at 2.05 Å, corresponding to the (111) plane of *fcc*-Co (JCPDS: 00–015–0806) in the nanoparticle core, and a longer spacing of 2.24 Å at the nanoparticle surface, which can be attributed to the (111) plane of Pd-associated *fcc* phases, namely *fcc*-CoPd (JCPDS: 03–065–6075) or *fcc*-Pd (JCPDS: 00–001–1201) (Figure 2g).

In contrast to the metallic cobalt bulk of the NPs as identified by PXRD and XAFS, X-ray photoelectron spectroscopy (XPS) detected quantities of oxidized cobalt at the surface and subsurface. However, the thick encapsulating carbon layer resulted in low signal intensity (see SI section S5, Figure S5.1). In the bimetallic sample, the palladium was fully metallic in this region. The quantification of atomic ratios obtained from the analysis of XPS regions (Co 2p*_3/2_* and Pd 3d) revealed a lower Co:Pd ratio than obtained by XRF (82:18 compared to 90:10), but higher to that calculated by PXRD for the CoPd alloy phase (33:67), indicating both slightly surface enrichment of palladium. These findings align with HRTEM images, which indicate that palladium is primarily located on the surface of the nanoparticles. The satellite features in the C 1s region, located several eV above the main peak (ca. 291 eV), are typically associated with a high concentration of sp^2^ hybridization, indicating the presence of graphitic carbon (Figure S5.1c).

Raman spectra of both catalysts **Co@C** and **CoPd/Co@C** (see SI section S6, Figure S6.1) show two major peaks at 1351 cm^–1^ and 1594 cm^–1^ for **Co@C** and 1355 cm^–1^ and 1589 cm^–1^ for **CoPd/Co@C**, associated with D (local defects and disorder) and G (sp^2^-bonded carbon atoms) bands respectively, which are characteristic of carbon-based materials.^42^ In both catalysts, the higher contribution of the D indicates a greater number of structural defects (A_D_/A_G_ = 2.73 for **Co@C** and A_D_/A_G_ = 2.91 for **CoPd/Co@C**). These defects presumably facilitate the interaction between reaction gases and the active metal species, thereby improving the catalyst’s stability during magnetically induced catalysis (*vide infra*).

The magnetic properties of **Co@C** and **CoPd/Co@C** were determined using Vibrating Sample Magnetometry (VSM), applying a magnetic field from −3 to 3 T at 5 and 300 K. From the obtained hysteresis loops, it is possible to determine the values of saturation magnetization (M_S_), remanent magnetization (M_R_) and coercive field (H_C_) (Figure 2i, j; see Table S7.1). The M_S_ values obtained for **Co@C** are 172 A·m^2^·kg_Co_^–1^ at 5 K and 165 A·m^2^·kg_Co_^–1^ at 300 K, which are slightly higher than those obtained for a material also based on Co NPs encapsulated in carbon published in a previous work (Co@HDA·HCl/C; M_S_ of 120–130 A·m^2^·kg_Co_^–1^).^29^ In the case of **CoPd/Co@C**, it exhibits lower M_S_ values (137 A·m^2^·kg_Co_^–1^ at 5 K and 135 A·m^2^·kg_Co_^–1^ at 300 K) likely due to the inclusion of Pd, which is a nonmagnetic material. Interestingly, neither catalyst exhibits a significant contribution from *exchange bias* in the hysteresis loops (see zoomed region in Figure 2i, j), suggesting that no significant oxide layer is present on the surfaces of **Co@C** and **CoPd/Co@C** NPs, which was also confirmed by XPS and XAS (*vide supra*).

Finally, the heating capacity of **Co@C** and **CoPd/Co@C** was estimated by determining their specific absorption rate (SAR) via calorimetry using a previously reported procedure (see SI, section S7).^20,43,44^ The SAR values were obtained in the solid state by applying an alternating magnetic field with a frequency of 93 kHz and different field amplitudes (0 to 47 mT). As can be seen in Figure 2k, both materials start to heat up at 14 mT, reaching maximum values of 144 W·g^–1^ for **Co@C** and 69 W·g^–1^ for **CoPd/Co@C** at 47 mT. Nonetheless, both SAR values are high enough to reach elevated temperatures in the presence of an oscillating magnetic field (*vide infra*).

### Catalytic Performance Results

**Co@C** and **CoPd/Co@C** catalysts were used as heating and catalytic agents in the magnetically induced hydrogenation of CO_2_ into CO in a continuous flow, employing a 1:3 molar mixture of CO_2_:H_2_ with a working flow of 32 mL·min^–1^. For the magnetically induced reactions, an AC magnetic field oscillating at 320 kHz with a maximum of 2 kW power was used (see SI section S1). As shown in Figure 3a, **Co@C** seems to be completely selective toward producing CO across the entire range of the applied field amplitudes without observing any methane as a secondary product. The figure also shows how CO_2_ conversion increases as the applied magnetic field increases. For example, at 40 mT, the conversion is only 3.9%. In contrast, when we increase the applied magnetic field to 53 mT, the conversion progressively increases to 44.8%, achieving almost complete selectivity for the RWGS product in both cases (>99% CO). At the maximum field amplitude of the equipment, 63 mT, **Co@C** achieved a maximum conversion of 61.1% (see SI section S8, Table S8.1). The local temperature of the system (T_local_), measured with an ultrafine platinum thermocouple and corroborated with an IR pyrometer, also increases with the applied magnetic field, reaching a maximum value of 476 °C at 63 mT. Interestingly, the conversion obtained at 63 mT (61.1%) is higher than the equilibrium conversion estimated (employing the Gibbs free energy minimization method) for 476 °C, which is 46.8% (see black stars in Figure 3a). This suggests that in the presence of the oscillating magnetic field, the surface temperature (T_surf_) of **Co@C** is higher than the T_local_ measured, as has already been observed in previous works.^26,30^

**Figure 3:**
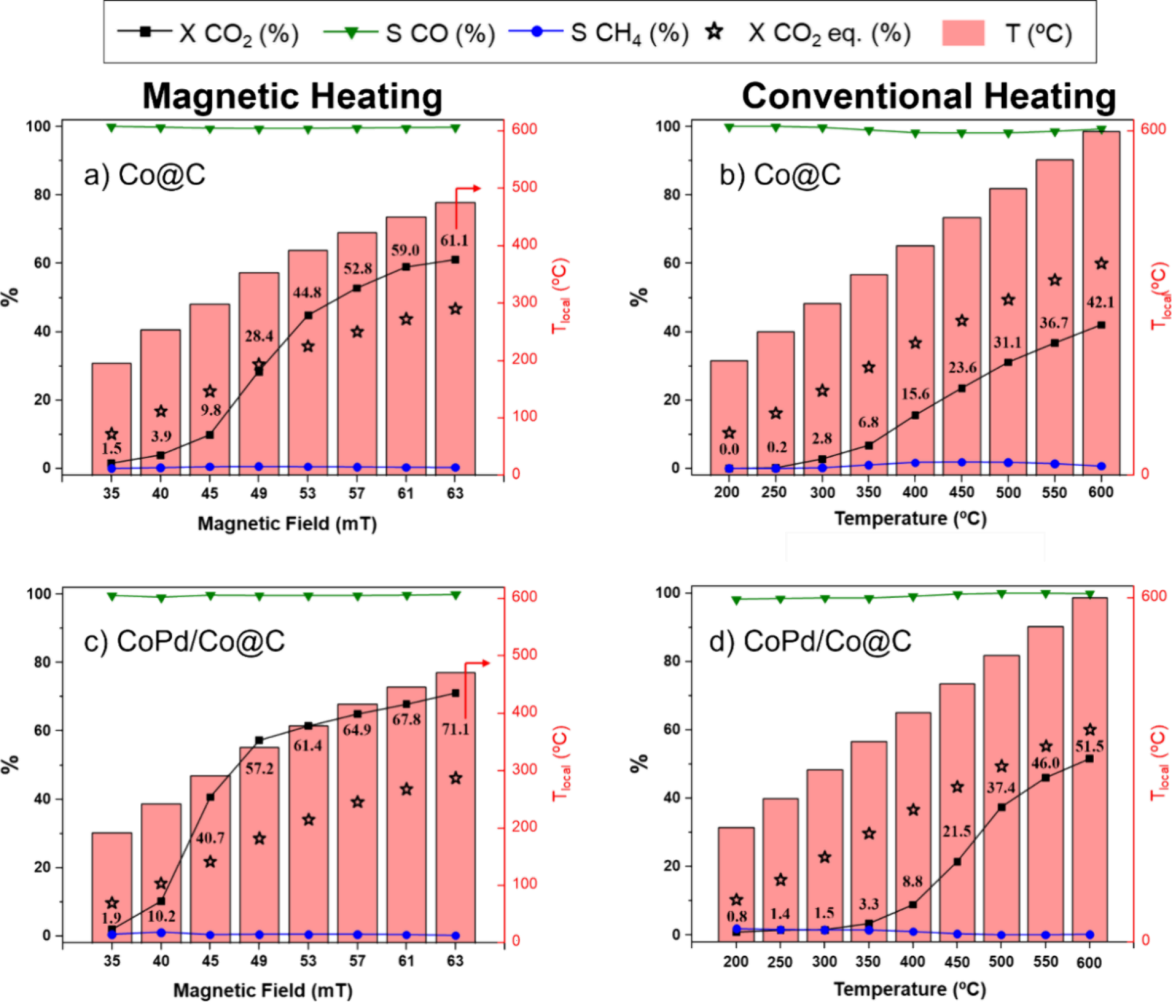
Magnetically (a, c) and conventionally (b, d) induced RWGS reaction for **Co@C** and **CoPd/Co@C** catalysts. Reaction conditions: 32 mL·min^–1^ CO_2_:H_2_ (1:3) (GHSV = 93.200 mL·h^–1^·g_metal_^–1^), *P* = 1 bar. X = conversion, and S = selectivity. Theoretical equilibrium conversion for CO_2_, estimated as a function of temperature, is represented by black stars.

To corroborate that the surface temperature of **Co@C** is higher than the one measured (T_local_), the catalyst performance was also tested in the RWGS reaction using conventional heating via a homemade oven (maximum power of 0.85 kW) with a maximum working temperature of 600 °C. As observed in Figure 3b, no significant conversions (below 10%) were observed for the conventionally heated **Co@C** until temperatures of 300–350 °C. The conversion continues to increase almost linearly until the maximum temperature of 600 °C; at this point, a conversion of 42.1% is achieved with a selectivity >99% toward CO. It is important to note that, unlike during magnetic induction heating in Figure 3a, the theoretical equilibrium conversion was not exceeded at any time using conventional heating. Additionally, comparing the maximum conversion obtained through magnetic induction heating and conventional heating (61.1% and 42.1%, respectively), it can be assumed that the T_surf_ when **Co@C** is magnetically induced at 63 mT is higher than 476 °C, probably exceeding 600 °C (see SI section S8, Table S8.2).

Motivated by the obtained results, we also tested the catalytic activity of **CoPd/Co@C** catalyst, by using the same catalytic conditions described above (GHSV: 93.200 mL·h^–1^·g_metal_^–1^ or 42.1 min^–1^; molar ratio CO_2_:H_2_ 1:3). As shown in Figure 3c, like **Co@C**, the selectivity for CO production with **CoPd/Co@C** is complete (>99%), and the conversion increases with the applied magnetic field, reaching a maximum conversion of 71.1% at 63 mT with a T_local_ of 471 °C (see SI section S8, Table S8.1). Compared to the literature, **CoPd/Co@C** heated by magnetic induction appears to be one of the most active catalysts for the RWGS reaction (see SI section S9, Table S9.1), reporting a space-time-yield (STY) for CO production of ca. 16.400 mL·h^–1^·g_metal_^–1^. Moreover, by increasing the flow rate up to 200 mL·min^–1^, the STY of CO can be increased until a maximum value of 46.300 mL·h^–1^·g_metal_^–1^ (see SI section S8, Figure S8.1), which is the highest value reported to date for magnetically induced RWGS reaction. Reference catalysts, such as Cu/ZnO/Al_2_O_3_ (CZA) or Pd/C (10 wt %), have been tested under the same catalytic conditions (GHSV: 93.200 mL·h^–1^·g_metal_^–1^), demonstrating their excellent performance in conventionally heated RWGS (see SI section S8, Table S8.3). Both catalysts achieve a similar conversion of around 55%, with CZA being fully selective for CO production, while Pd/C also generates CH_4_ as a reaction product (with a CH_4_ selectivity of 25.3%). However, since neither of these catalysts are magnetic, they are not active in magnetically induced RWGS. In contrast, **CoPd/Co@C**, which is magnetically active, achieves a higher conversion of 71.1%, making it more active than these reference catalysts under magnetic induction conditions.

Interestingly, at magnetic field amplitudes below 40 mT, the CO_2_ conversion with **CoPd/Co@C** is very low, not exceeding 10.2%, with a T_local_ of 243 °C. However, increasing the magnetic field to 45 mT causes a sharp increase in conversion, reaching a value of 41.7% with a T_local_ of 292 °C (see Table S8.1). Since the difference in T_local_ between 40 and 45 mT is not large (49 °C), the significant improvement in conversion must be due to a greater discrepancy in the surface temperature of the CoPd NPs, which is well above the measured T_local_, and appears necessary to activate the catalyst to increase the CO_2_ conversion substantially. Furthermore, when the magnetic field is increased beyond 45 mT, the obtained conversion exceeds the theoretical equilibrium conversion. To corroborate this theory, the activity of **CoPd/Co@C** in CO_2_ hydrogenation under conventional heating was compared. As shown in Figure 3d, to achieve a similar conversion to that obtained at 40 mT (10.2%), a temperature of around 400 °C must be applied, much higher than the measured T_local_ (243 °C). To achieve a conversion close to that obtained at 45 mT (41.7%, T_local_ 292 °C), a temperature close to 550 °C is needed. This example corroborates that **CoPd/Co@C** needs to reach a surface temperature above 500 °C to achieve the outstanding conversion observed, further highlighting the higher surface temperature attained in **CoPd/Co@C** when magnetically induced. On the other hand, when comparing the results obtained from **Co@C** and **CoPd/Co@C** under conventional heating, the monometallic **Co@C** catalyst exhibits higher activity at temperatures below 450 °C. For example, at 400 °C, **Co@C** achieves a CO_2_ conversion of 15.6%, while **CoPd/Co@C** shows a slightly lower conversion of 8.8%, suggesting that at lower temperatures, **Co@C** is a more active catalyst than **CoPd/Co@C**. To further support this observation, CO_2_ temperature-programmed reduction (CO_2_-TPR) experiments were performed for both catalysts, revealing a similar trend to conventional heating catalysis. As shown in Figure S10.1 (see SI section S10), **Co@C** begins producing CO at around 350 °C, whereas **CoPd/Co@C** only becomes active in the RWGS at temperatures above 600 °C. These results confirm that **Co@C** is more active at lower temperatures, while **CoPd/Co@C** is more suitable for higher-temperature conditions. Furthermore, the CO_2_-TPR profiles provide further evidence that under magnetic induction heating, the actual surface temperature of the catalysts is significantly higher than the measured local temperature. In fact, **CoPd/Co@C** displays superior activity across the entire range of applied magnetic fields, indicating that the surface temperature of the MagNPs must exceed 450 °C during MIH.

As we mentioned, the electrification of the processes is critical from an energy-saving perspective. Comparing the energy consumption of magnetic induction heating with conventional heating reveals that the most relevant difference lies in heating times. For instance, to achieve a CO_2_ conversion of ca. 40% with **CoPd/Co@C**, the conventional electric oven must reach a temperature of 550 °C (42.3% conversion), which takes 27.5 min (20 °C/min). If the power consumption of the electric oven is 0.85 kW·h, it is estimated that the heating ramp consumes 390 W·h. However, through MIH, **CoPd/Co@C** can achieve the exact conversion (41.7%) by applying a magnetic field of 45 mT (0.81 kW·h). As shown in Figure S11.1 (see SI section S11), **CoPd/Co@C** achieves the same conversion in less than 2 min, thanks to the fast heating rate of MIH, resulting in an energy cost of just 27 W·h during the heating ramp. Thus, MIH is 15 times more energy-efficient during the heating ramp than conventional heating under analogous operating conditions. In addition, the system demonstrates rapid heating capabilities across different magnetic fields, allowing for precise control of temperature variations in a short time (see SI Section 11, Figure S11.2). Furthermore, comparing the results obtained with those reported to date for the magnetically induced RWGS reaction, it is evident that **CoPd/Co@C** is the most energy-efficient catalyst for CO production. As shown in Table S9.1 (see SI section S9), very few catalysts have been reported in the literature for magnetically induced RWGS. However, **CoPd/Co@C** is up to six times more energy-efficient than the best catalyst reported to date (170.4 mL_CO_/kW·h for **CoPd/Co@C**vs 27.8 mL_CO_/kW·h for Co@Ni@C).^39^ Furthermore, increasing the flow rate to 200 mL·min^–1^ while using the same magnetic equipment (2 kW of maximum power) boosts the process efficiency from 170.4 to 478.5 mL_CO_/kW·h, without incurring additional energy costs.

To further corroborate the theory that the T_surf_ of both catalysts is higher than the T_local_ measured during magnetically induced catalysis, the activation energy (Ea) of **Co@C** and **CoPd/Co@C** was calculated through the Arrhenius equation using conventional heating (Figure 4a; and see SI section S8, Table S8.4 and Figures S8.2 and S8.3). In this way, and assuming both conventional and magnetic heating follow the same reaction mechanism, the estimated T_surf_ values at different magnetic fields can be determined by interpolating the values of the kinetic constants obtained through magnetic induction heating.^26,30^ Thus, the calculated Ea for **Co@C** is 36.1 kJ·mol^–1^, while for **CoPd/Co@C,** it is 30.9 kJ·mol^–1^. The lower Ea observed for **CoPd/Co@C** is consistent with the previously obtained catalytic results, as the bimetallic CoPd-based catalyst demonstrates greater activity in the RWGS reaction. Then, by introducing the initial rate values obtained through magnetically induced catalysis into the conventionally heated Arrhenius plot (see SI section S8, Table S8.5 and Figure S8.4), we were able to estimate the T_surf_ of both catalysts during the magnetically induced RWGS reaction (Figure 4c). Specifically, for **Co@C**, the estimated surface temperature of the nanoparticles increases linearly over the entire range of the magnetic field, reaching a maximum estimated T_surf_ value of 840 °C at 63 mT. In the case of **CoPd/Co@C**, the temperature of the surface of CoPd NPs also increases with the magnetic field applied, with the maximum estimated T_surf_ being 808 °C. This lower estimated T_surf_ for **CoPd/Co@C** is expected since Pd is nonmagnetic, translating into lower SAR values and, therefore, a lower heating capacity (*vide supra*). Nevertheless, despite reaching a lower estimated T_surf_, **CoPd/Co@C** has proven to be the most active catalyst for magnetically induced RWGS.

**Figure 4:**
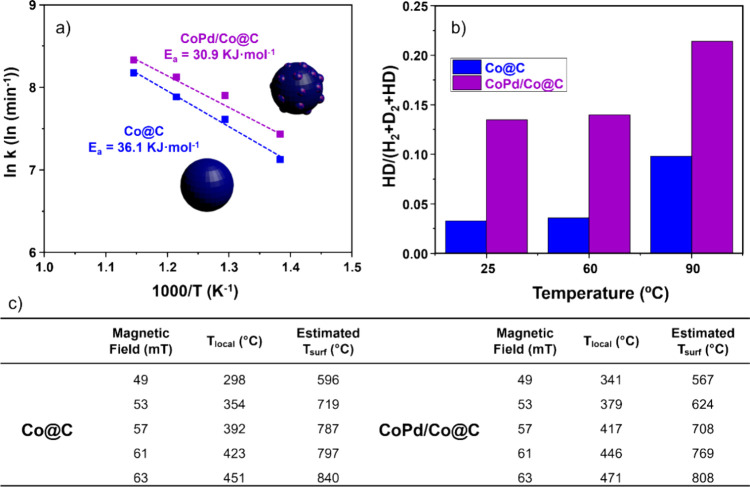
(a) Arrhenius plots for the RWGS heated conventionally over **Co@C** (blue) and **CoPd/Co@C** (violet). (b) Increment of HD mass signal during H/D exchange experiments at r.t., 60 °C, and 90 °C using **Co@C** and **CoPd/Co@C** as catalysts (prereduced by heating at 120 °C with a H_2_ flow of 10 mL/min). (c) Estimated T_surf_ for **Co@C** and **CoPd/Co@C** when using MIH at different field amplitudes. T_local_ was measured by a platinum thermocouple (type K temperature probe) and corroborated with an IR pyrometer.

Based on the higher conversion achieved by the **CoPd/Co@C** catalyst compared to its monometallic counterpart **Co@C** in the RWGS reaction (71.2% vs 61.1%), it is logical to attribute this improvement to the inclusion of Pd atoms in the system. As said before, it is widely known that Pd can more easily activate the hydrogen molecule (i.e., homolytic bond breaking).^40^ If we look at the T_local_ reached by **CoPd/Co@C** at 63 mT, we can observe that it is lower than that achieved with **Co@C** under the same magnetic field (476 vs 471 °C). This is logical when considering the SAR values measured (see Figure 2k), where **Co@C** shows a higher SAR value of 144 W·g^–1^, compared to 69 W·g^–1^ for **CoPd/Co@C**. Interestingly, although **Co@C** reaches a higher T_local_, it is less active in the RWGS reaction, indicating that the small incorporation of Pd atoms into the Co NPs enhances their activity.

Finally, the stability of both catalysts was explored under the standard reaction conditions described above (GHSV: 93.200 mL·h^–1^·g_metal_^–1^or 42.1 min^–1^, molar ratio CO_2_:H_2_ 1:3). As shown in Figure 5a, at initial reaction times and with the application of 63 mT, **Co@C** exhibits a conversion of 60.8% with a local temperature of 472 °C. However, as the reaction time increases, a clear decrease in activity can be observed, with the conversion rate dropping by 10% after 100 h of reaction (to 51.1% of conversion). Interestingly, the measured temperature decreases over time, falling from 472 to 452 °C. This decrease in activity is possibly due to the partial oxidation of **Co@C** by the action of the water formed in the RWGS, which we have corroborated through VSM and SAR measurements (see SI section S12, Figure S12.1). Partial oxidation was confirmed by the presence of a minor *exchange bias* observed in the hysteresis loops at 5 K after a field cooling in the presence of a μ_0_H of 3 T (see zoomed region in Figure S12.1a), which is characteristic of the coupling between ferromagnetic and antiferromagnetic layers.^45^ Therefore, this oxidation causes a reduction in its magnetic properties (SAR_before_ = 144 W·g^–1^vs SAR_after_ = 68 W·g^–1^) and, consequently, in the local temperature achieved. The oxidation of the Co NPs was also evidenced through the HRTEM of the material after catalysis, where planes due to the formation of Co_3_O_4_ on the surface of the nanoparticle can be observed (see SI section S13, Figure S13.2). The partial oxidation of Co NPs was experimentally corroborated by a catalytic reactivation step, where, after exposing the **Co@C** under N_2_ at 300 °C for 1 h, it recovered and even surpassed its initial catalytic activity (68.2% conversion). Thus, **Co@C** has proven to be stable for more than 3 catalytic cycles of reaction-activation-reaction (Figure 5a), without a considerable increase in particle size or loss of crystallinity (see SI section S13, Figure S13.2).

**Figure 5:**
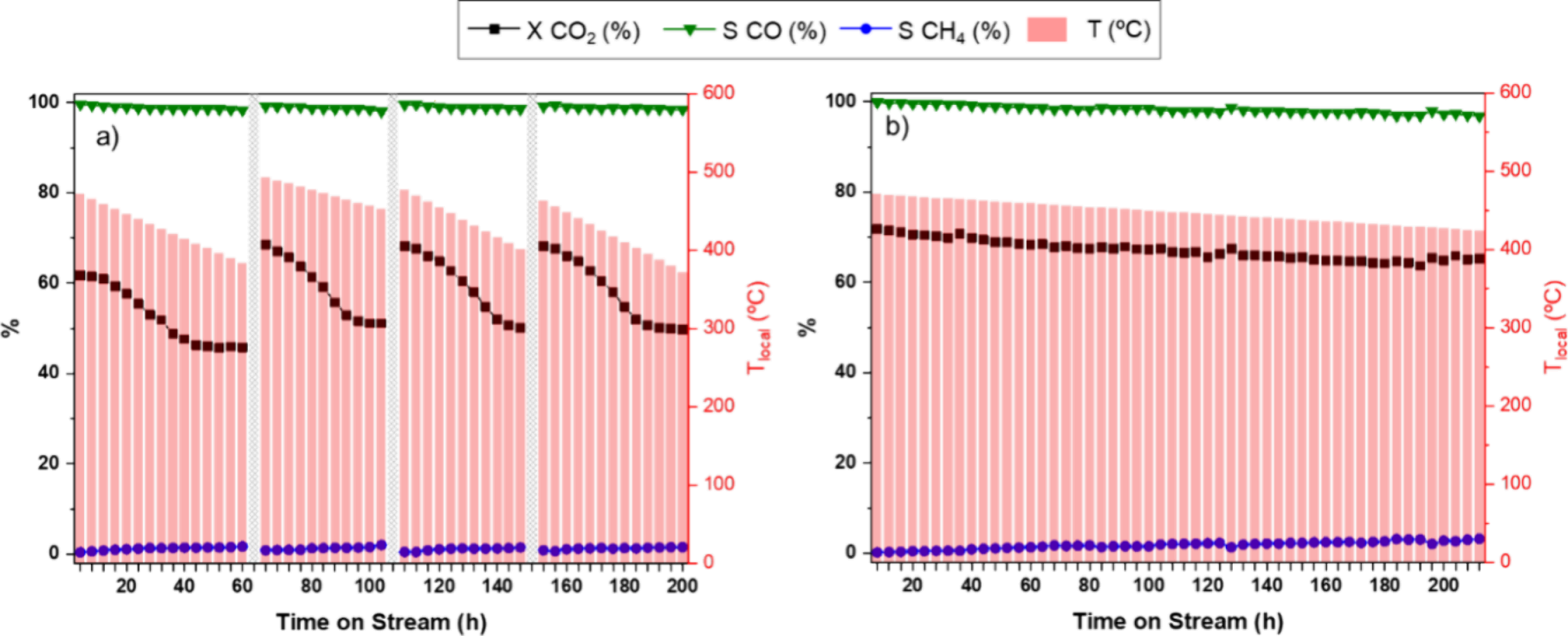
Catalytic performance of (a) **Co@C** and (b) **CoPd/Co@C** over time in the magnetically induced RWGS reaction (63 mT). Reaction conditions: 32 mL·min^–1^ CO_2_:H_2_ (1:3) (GHSV = 93.200 mL·h^–1^·g_metal_^–1^), *P* = 1 bar. X = conversion, and S = selectivity. Dashed gray bars represent the activation process of the catalyst (N_2_ for 1 h at 300 °C).

Regarding **CoPd/Co@C**, it behaves differently from its monometallic counterpart. As shown in Figure 5b, **CoPd/Co@C** exhibits an initial conversion of 71.8%, which only slightly decreases to 65.3% after more than 200 h of reaction, without any need for intermittent reactivation. This higher stability can be explained by the fact that CoPd NPs are less oxidized by the water formed during the RWGS, as no significant decrease in the measured local temperature is observed. This may be attributed to a possible role of Pd in enhancing the reduction of Co species that are oxidized by water. The study of the magnetic properties of **CoPd/Co@C** after catalysis (see SI section S12, Figure S12.2) revealed that the SAR values do not decrease as drastically as they do for **Co@C** (SAR_before_ = 69 W·g^–1^vs SAR_after_ = 62 W·g^–1^). That is why performing activation-reactivation cycles as required for **Co@C** is unnecessary.

Postreaction characterization was performed by ex situ XAFS, XPS, TPR and HRTEM (see SI sections S3, S5, S10 and S13, respectively). Regarding EXAFS analysis, the coordination number of the Pd–Co path in the alloyed phase at the Pd K edge increases after the reaction, whereas that of metallic Co^0^ decreases (Table S3.4). In the XPS data, the Pd signal intensity decreases significantly after the reaction (Figure S5.1), and the quantification of atomic ratios from the Co 2p*_3/2_* and Pd 3d regions reveals a higher Co:Pd ratio (82:18 before the reaction vs 94:6 after), indicating cobalt surface enrichment. These findings suggest that Pd partially migrates from the surface toward the NP core during reaction, accompanied by a slight migration of Co from the metallic Co^0^ phase to the alloyed CoPd phase. Linear combination fitting of the XANES spectra, performed before and after the reaction, revealed a 15% increase in oxidized species for the monometallic catalyst, compared to only a 6% increase for the bimetallic catalyst. This cobalt oxidation is unlikely to be due to the formation of carbides, as evidenced by XPS, and may be attributed instead to Co’s capacity to dissociate CO_2_, particularly when present as larger crystallites.^46^ Temperature-programmed reduction (TPR) analysis of both systems (see SI Section 10, Figure S10.2) further support this, showing that after magnetic catalysis, **Co@C** exhibits a significantly higher degree of oxidation compared to **CoPd/Co@C**, which does not display a distinct reduction peak associated with any oxidized active species. Therefore, these findings suggest that the presence of Pd inhibits the surface oxidation of the nanoparticles.^47^

The RWGS reaction involves the adsorption and activation of CO_2_ and H_2_ on the catalyst surface, followed by their transformation and desorption of CO and H_2_O. Therefore, to understand why one catalyst exhibits higher activity than another, we investigated in detail the adsorption–desorption behaviors of these molecules on both **Co@C** and **CoPd/Co@C** catalysts. In Figure 4b, H/D isotopic exchange for both catalysts is presented. Across the entire temperature range studied (25, 60, and 90 °C), the HD mass signal is consistently higher for **CoPd/Co@C** than for **Co@C** indicating that the Pd-based catalyst dissociates H_2_ faster.^48^ Additionally, CO_2_ and CO temperature-programmed desorption (TPD) analyses were performed. The CO_2_-TPD profiles of both magnetic catalysts reveal similar desorption features, with distinguishable weak and strong adsorption peaks around 100 and 500–600 °C,^49^ respectively (see SI section S10, Figure S10.3). However, a clear difference in total CO_2_ uptake is observed. The palladium incorporation into Co NPs significantly enhances CO_2_ adsorption capacity, leading to a notable 22% increase in CO_2_ uptake. On the other hand, as expected from the high selectivity to CO (>99%) shown by both catalysts, CO-TPD experiments did not reveal any remarkable differences between the two systems, indicating that both mono- and bimetallic catalysts effectively desorb CO (see SI section S10, Figure S10.4). Based on these results, the rate-determining step of the RWGS reaction magnetically induced remains inconclusive, both CO_2_ and H_2_ adsorption as well as their activation or surface reactions may be involved, while CO desorption is not as both catalysts exhibit similar CO desorption behavior. However, previous studies have shown that MIH promotes an electronic phenomenon known as the skin effect, which leads to an accumulation of electrons on the catalyst surface, leading to the formation of an HCOOH-based reaction intermediate.^50^ What is evident is that the enhanced H_2_ dissociation rate on **CoPd/Co@C** plays a key role in improving the catalyst stability, as the presence of palladium prevents surface oxidation of cobalt in the presence of water, thereby eliminating the need for repeated reaction-activation-reaction cycles that are required for the **Co@C** catalyst.

On the other hand, the partial oxidation of Co highlights the dual functionality of carbon encapsulation. It enhances the stability of these MagNPs by preventing agglomeration, ensuring they retain both their catalytic activity and heating capacity. At the same time, the carbon-encapsulation remains permeable enough to allow the reactant to reach the nanoparticle surface. This outstanding result is one of the few reported examples where magnetic induction heating is used over extended periods (>200 h) for the RWGS reaction, making **CoPd/Co@C** a viable catalyst under industrial conditions.

## Conclusions

This study demonstrates the feasibility and advantages of using MIH as a novel and electrified technology to conduct the RWGS reaction, offering a promising pathway for CO_2_ conversion. Two novel catalysts were synthesized through the pyrolysis of a Co-based MOF: carbon-encapsulated nondoped cobalt nanoparticles (**Co@C**) and palladium-doped cobalt nanoparticles (**CoPd/Co@C**). The evolution of metallic phases during the pyrolysis process was monitored using *in situ* PXRD and XAS, confirming that the MOF’s collapse during pyrolysis led to the formation of well-defined MagNPs, with sizes of 15 nm for **Co@C** and 17 nm for **CoPd/Co@C**. The selected pyrolysis conditions promoted the formation of a well-defined carbon shell encapsulating the metallic nanoparticles, with thicknesses of ca. 3 nm in both cases. This encapsulation prevented agglomeration and preserved both the catalytic activity and heating capacity of the catalysts. In the bimetallic catalyst, incorporating Pd led to the formation of small CoPd nanoparticles, approximately 7 nm in size, on the surface of the Co core. These nanoparticles, resulting from the alloying of Co and Pd, exhibited a defined composition of Co_0.33_Pd_0.67_.

Under magnetically induced RWGS conditions, both catalysts exhibited outstanding activity and selectivity for syngas production, with CO yields of 61.1% for **Co@C** and 71.2% for **CoPd/Co@C** at 63 mT, 2 kW, and 320 kHz. Notably, **CoPd/Co@C** achieved the highest CO production efficiency reported to date for magnetically induced RWGS, reaching 478.5 mL_CO_/kW·h. The localized and direct heating provided by MIH allowed these catalysts to operate at lower bulk temperatures and with greater energy efficiency than conventional heating methods. This is primarily attributed to the rapid heating and higher temperatures at the MagNPs surface. The T_surf_ of **Co@C** and **CoPd/Co@C** during the magnetically induced catalysis was estimated by establishing a correlation between the apparent kinetic constant and the temperature using conventional heating. This kinetic approach estimated a T_surf_ of 840 °C for a 61.1% conversion for **Co@C** and 808 °C for a 71.2% conversion for **CoPd/Co@C**.

In addition to their efficiency, the catalysts demonstrated remarkable stability. While **Co@C** showed good stability, requiring reaction-activation-reaction cycles after approximately 50 h, **CoPd/Co@C** maintained its performance for over 200 h without significant deactivation or the need for reactivation, highlighting the crucial role of Pd-doping in enhancing stability by inhibiting the oxidation of Co. Furthermore, the carbon encapsulation also contributes to the stability of both catalysts by preventing the agglomeration of the MagNPs at high temperatures, ensuring they retain both their catalytic activity and heating capacity. This stability, combined with the high activity of the catalyst, establishes MIH-conducted RWGS as a highly promising technology for energy-intensive reactions such as CO_2_ reduction. The process not only minimizes energy consumption but also achieves exceptional efficiency, aligning with global objectives for sustainability and cost reduction.

## Materials and Methods

### General Considerations and Starting Materials

All chemicals were purchased from Merck or ABCR, and used as received. The metallic Co precursor ([Co_4_O_4_(OAc)_4_(py)_4_]) was synthesized according to published procedures.^51^

### High-Resolution Transmission Electron Microscopy (HRTEM)

TEM and HRTEM micrographs of **Co@C** and **CoPd/Co@C** nanoparticles were obtained after suspending a drop of the corresponding material in EtOH on a copper grid. HRTEM analyses were performed at the Electron Microscopy Service of the Universitat Politècnica de València (UPV) using a JEOL 2100F microscope operated at 200 kV in transmission (TEM) and scanning transmission (STEM) modes. EDX and STEM images were obtained using a darkfield (DF) detector. Particles were manually measured with ImageJ software, and interplanar spacing and fast Fourier transform (FFT) treatments were performed with Digital Micrograph (version 3.7.4). The average particle size was determined by manually analyzing enlarged micrographs and measuring the size of particles on a specified grid.

### Scanning Electron Microscopy of Field Emission (FESEM)

FESEM images were acquired using an Ultra 55 (Zeiss), operating at 2.0 kV, using powder samples of 2D-CoMOF/C and Pd/2D-CoMOF/C prepared on a sample holder with an S4 double-sided adhesive tape for the dispersion of the sample. Samples were coated with carbon to avoid the charging effect.

### Raman Spectroscopy

For Raman spectra measurement, an excitation wavelength of 514 nm was used on a Renishaw inVia Raman spectrometer equipped with a CCD detector and a Leica microscope. The powder samples were deposited on an Al support and measured in the region between 0 and 3000 cm^–1^ with a resolution of <4 cm^–1^. A total of 20 acquisitions were made for each spectrum.

### X-ray Fluorescence (XRF)

X-ray fluorescence spectra of the catalysts were recorded in a Zetium XRF spectrometer. Before measuring the catalysts, the calibration curve was adjusted to the predicted concentration of the analyte using commercial standards.

### X-ray Photoelectron Spectroscopy (XPS)

The XPS spectra of **Co@C** and **CoPd/Co@C** were recorded using a Kratos AXIS Supra spectrometer equipped with a charge neutralizer and a monochromated Al Kα excitation source (16.7 eV) with a step size of 0.1 eV and a pass energy of 20 eV. Spectra were analyzed using CasaXPS software (version 2.3.18). Binding energy (BE) values were referenced to the C 1s peak at 284.8 eV. For the metallic core lines, asymmetry was defined using the LA (α, β, m) function, where α and β specify the tail spread on either side of the Lorentzian component, and the parameter m indicates the width of the Gaussian used to convolute the Lorentzian curve. For the remaining components, a Gaussian (Y%) – Lorentzian (X%) mix, defined in CasaXPS as GL­(X), was applied. Based on the referenced literature, the values LA­(1.2, 5, 5) and GL(30) were used.^47^

### Temperature-Programmed Reduction (TPR)

The TPR analyses were performed using a Micromeritics AutoChem 2910 system with a thermal conductivity detector (TCD). Before analyzing the 50 mg samples of **Co@C** and **CoPd/Co@C**, they were pretreated at room temperature in flowing helium (He) at a rate of 10 mL/min for 20 min. Subsequently, the samples were heated from 25 to 600 °C at a rate of 10 °C/min in a flow of 50 mL/min of diluted hydrogen (H_2_) in argon (Ar) (10% H_2_ volume concentration).

### Temperature-Programmed Reduction (TPR) of CO_2_

CO_2_-TPR of **Co@C** and **CoPd/Co@C** were performed using a quartz tubular reactor connected to a Balzer QMC 220M1 mass spectrometer. For each measurement, 50 mg of catalyst was exposed to a gas mixture of CO_2_:H_2_ in a 1:3 ratio, flowing at 32 mL/min. The samples were heated at a constant rate of 10 °C/min up to a maximum temperature of 800 °C. Throughout the experiment, the mass spectrometer continuously monitored the evolution of CO_2_, H_2_, H_2_O and CO to assess the reduction behavior of the catalysts under reaction conditions.

### Temperature-Programmed Desorption (TPD) of CO_2_ and CO

TPD of CO_2_ and CO were performed using a quartz reactor connected to a mass spectrometer Balzer (QMC 220M1). For the measurements, 50 mg of each sample was degassed at room temperature in a flow of 10 vol % CO_2_ or CO in Ar (20 mL/min) during 1 h. After the adsorption, the temperature was increased to 600 °C (10 °C/min), maintaining the Ar flow. CO_2_ and CO were followed by MS.

### Gas Chromatography Coupled to Mass Spectrometry (GC-MS)

GC-MS analyses were performed using an Agilent 8890 Gas Chromatograph (J&W HP-PLOT Q GC Column, 30 m, 0.32 mm, 20 μm; HayeSep Q, 80/100 mesh, 1m 1/8inch OD, 2 mm ID, stainless steel; J&W GC packed column, 2.44m, 1/8inch OD, 2 mm ID; and a MolSieve 5A packing, mesh size 60/80, preconditioned) with a TCD detector coupled to a Pfeiffer Vacuum GSD 350 O1Mass Spectrometer. Reactants, conversions, product yields, and selectivities were calculated by conducting a C balance on the chromatograms. Peak areas were adjusted using their response factors obtained after calibrating the TCD detector.

### Vibrating-Sample Magnetometer (VSM)

Magnetic measurements were conducted using the VSM equipment Quantum Device PPMS Evercool II. The VSM analysis was performed on compacted powder samples that were prepared and sealed in an argon atmosphere. The hysteresis loops were measured for magnetization vs magnetic field at 300 and 5 K, using an external field of up to ± 3 T.

### X-ray Absorption Spectroscopy (XAS) and X-ray Diffraction (XRD)

*Ex situ* X-ray absorption spectroscopy was performed at the ALBA Synchrotron (Cerdanyola del Vallès, Barcelona, Spain) on the BL16-NOTOS beamline through proposal 2023097780, and at the Diamond Light Source (Didcot, UK) on the B18 beamline through the UK Catalysis Hub Block Allocation Group Access (experiment SP34632–4). Data were collected at the Pd and Co K-edges in either fluorescence or transmission mode, and a minimum of three spectra were merged per sample. The samples (powders) were mixed with boron nitride and prepared as circular pellets using uniaxial pressing. *Ex situ* powder diffraction was also collected on the BL16-NOTOS beamline through proposal 2023097780 at either 13 keV (0.9537Å) or 23 keV (0.5393Å), depending on whether the Co K edge or Pd K edge was being measured. The samples were measured in 1.5 mm ID capillaries, some of which had different wall thicknesses resulting in different contributions of quartz in the diffraction patterns. Some data were converted to 2θ values for 23 keV during data processing for a simpler comparison to other literature data.

In the *in situ* experiments, samples were packed into a custom-made capillary system designed and constructed by ALBA. 2D-CoMOF/C or Pd/2D-CoMOF/C samples were placed inside a quartz capillary, immobilized between two quartz wool plugs. Nitrogen gas was flowed through the capillary at 20 mL/min, while the temperature (increased at 25 °C/min up to 800 °C, with a 2-h hold) was controlled by a hot air gun. The capillary was rocked in place during heating to improve sample averaging by allowing differently oriented grains to contribute to the pattern and to prevent the buildup of hotspots. XAS data was collected in transmission at the Pd K edge during isothermal sections after each 100 °C increase, with 3 spectra merged at each temperature. PXRD was collected at 23 keV continuously during the ramp, though only data each 50 °C are shown.

Analysis and fitting of XAS data were performed using the Strawberry Demeter packages Athena and Artemis for XAS,^52^ A Co^0^ and Pd^0^ foils were measured on both beamlines, and used to calculate the S_0_^2^ value for EXAFS fitting. For the PXRD data, wavelength and sample–detector distance were corrected during preprocessing, and Rietveld refinements were performed using GSAS II,^53^ fitting the broad carbon peak at a low angle as part of the background (chebyschev^–1^ function).

### Synthesis of Co@C and CoPd/Co@C Catalysts

#### Co@C

The monometallic carbon-based catalyst has been synthesized following a two-step procedure. In the first step, one equivalent of [Co_4_O_4_(OAc)_4_(py)_4_] (0.46 mmol) and 4 equiv of 2,2′-bipyridine-4,4′-dicarboxylic acid (bda) (1.84 mmol) were dissolved in 10 mL of pyridine. Then, carbon powder (VULCAN XC72R, 100 mg) was added to the solution and mixed with 8 equiv of trifluoroacetic acid (TFA). The resulting solution was introduced into a stainless-steel autoclave and heated at 150 °C for 9 days under autogenous pressure and dynamic conditions. Once cooled to room temperature, the solution was filtered, and the powder was washed with acetone to remove the remaining pyridine solvent molecules. The obtained solid was dried under vacuum, after which it was ground to a fine powder. At this stage, the sample was labeled as 2D-CoMOF/C. In the second step, the material was transferred into a quartz reactor and placed in a vertical oven. The sample was pyrolyzed with a ramp rate of 25 °C/min and held at 800 °C for 2 h under nitrogen flow (20 mL/min). Finally, the sample was cooled to room temperature under a nitrogen stream. The average size of **Co@C** is 7.5 ± 4.0 nm. XRF analyses revealed an 11.1 wt % of Co.

#### CoPd/Co@C

The bimetallic carbon-based catalyst was synthesized following the same procedure, but the palladium salt was included in the first one-pot step. One equivalent of [Co_4_O_4_(OAc)_4_(py)_4_] (0.46 mmol) and 4 equiv of 2,2′-bipyridine-4,4′-dicarboxylic acid (1.84 mmol) were dissolved in 5 mL of pyridine. Moreover, 12.84 mg of Na_2_PdCl_4_ was dissolved in 5 mL of ethanol. Both solutions were mixed, and carbon powder (VULCAN XC72R, 100 mg) and 8 equiv of trifluoroacetic acid (TFA) were added to the resultant solution. This mixture was introduced into a stainless-steel autoclave and heated at 150 °C for 9 days under autogenous pressure and dynamic conditions. The solution was filtered, washed with acetone and grounded. Up to this point, the sample was labeled Pd/2D-CoMOF/C. Finally, the material was pyrolyzed following the same procedure used for the Co@C. The average size of CoPd/Co@C is 12.7 ± 6.5 nm. XRF analyses revealed a 10.3 wt % of Co and 2.2 wt % of Pd.

## Supplementary Material



## Notes

Additional characterization studies before and after catalysis (including *in situ* and *ex situ* combined XRD and XAS, FESEM, HRTEM-STEM, XPS, Raman spectroscopy, and SAR measurements); supporting results for magnetically induced catalysis; and state-of-the-art comparison (PDF)
