# Supplementary material for: Pd-Enhanced Carbon-Encapsulated Co Nanoparticles for Efficient Reverse Water–Gas Shift under Magnetic Induction Heating
Source: ACS Catal. 2025 May 20;15(11):9489–502. doi: 10.1021/acscatal.5c01232 (PMC12150269; doi:10.1021/acscatal.5c01232)
Supplement: Supplementary file 1 [file cs5c01232_si_001.pdf]

Supporting Information (SI)

Pd-Enhanced Carbon Encapsulated Co Nanoparticles for Efficient RWGS under Magnetic Induction Heating

Adrián García-Zaragoza,[a,†] José Luis del Rio-Rodríguez,[a,†] Christian Cerezo-Navarrete,[a] Silvia Gutiérrez-Tarriño,[a] M. Asuncion Molina,[bc] Lucy G. Costley-Wood,[bc] Jaime Mazarío,[d] Bruno Chaudret,[d] Luis M. Martínez-Prieto,[a,e] Andrew M. Beale[b,c,] and Pascual Oña-Burgos[a,*]*

[a] *ITQ, Instituto de Tecnología Química, Universitat Politècnica de València (UPV), Av. de los Naranjos S/N, 46022, Valencia, Spain.*

[b] *Department of Chemistry, University College London, 20 Gordon Street, WC1H 0AJ, UK.*

[c] *Research Complex at Harwell, Rutherford Appleton Laboratories, Harwell Science and Innovation Campus, Harwell, Didcot, OX11 0FA, UK.*

[d] *LPCNO, Laboratoire de Physique et Chimie des Nano-Objets, Université de Toulouse, CNRS, INSA,UPS, Toulouse 31077, France.*

[e] *IIQ, Instituto de Investigaciones Químicas (CSIC-Universidad de Sevilla), Avda. Americo Vespucio 49, 41092, Seville, Spain.*

**Corresponding authors: Pascual Oña-Burgos (pasoabur@itq.upv.es), Andrew M. Beale (andrew.beale@ucl.ac.uk).*

†Adrián García-Zaragoza and José Luis del Rio-Rodríguez contributed equally to this paper.

Table of Content

S1.	Catalytic reaction	3
S2.	Experimental catalytic setup	6
S3.	*In situ* and *ex-situ* combined XRD and XAS	7
S4.	FESEM	16
S5.	XPS	17
S6.	RAMAN	17
S7.	SAR measurement	18
S8.	Magnetically induced catalytic results	19
S9.	State-of-the-art catalysts for CO_2 reduction	24
S10.	Absorption studies	25
S11.	MIH operating in intermittent conditions	27
S12.	VSM	28
S13.	HRTEM and STEM images	29
	References	30

S1. Catalytic reaction

S1.1. Magnetically induced catalytic reaction

Catalytic experiments were performed in a continuous fixed-bed quartz tube reactor with an outer diameter of 12 mm and an inner diameter of 8 mm. The catalyst was deposited on a porous quartz frit located within the tubular reactor (see section S3, Figure S3.1). The quartz reactor was placed at the centre of a coil connected to an AC magnetic induction system (Ultraflex) of 2 kW (maximum power) oscillating at a frequency of 320 kHz with a root-mean-square (RMS) amplitude adjustable between 0 and 63 mT. The coil (manufactured by Ultraflex) consists of a copper solenoid with 6 turns with an inner diameter of 24 mm and a height of 35 mm.

The local temperature was measured using a platinum thermocouple (type K temperature probe) located at the same position as the catalytic bed outside the quartz reactor. The thermocouple's response was measured and corrected at different field amplitudes to avoid measurement errors. Furthermore, the local temperature was verified using an infrared pyrometer, without observing temperature differences exceeding 5 ºC.

For **Co@C** and **CoPd/Co@C**, the required amount of catalyst is introduced to achieve the same GHSV in the RWGS reaction, supplying a flow rate of 32 mL·min^{-1} with a CO_2:H_2 molar ratio of 1:3 (GHSV: 93.2 L·h^{-1}·g$_{metal}^{-1}$; 42 min^{-1}).

The GC analysis method was conducted using both pure gases and mixed gases with certified compositions. The response factor (FR_X) of each analyte was determined by injecting known amounts of analyte X into the chromatograph, using N_2 as an internal standard.

We can define the following equations:

$$A_X = \%X{\cdot}FR_X \quad , \quad \%X = \frac{A_X}{FR_X}$$

where A_X and A_{N_2} are the areas of the analyte X and molecular nitrogen, respectively. To obtain the FR_X of any substance, the amount of N_2 in the sample must be expressed. To do this, we divide the expression for $\%X_{in}$ by that for $\%N_{2,in}$ to then isolate FR_X, resulting in:

$$FR_X = \frac{A_X}{A_{N_2}} \frac{\%N_{2,in}}{\%X_{in}}$$

The percentage of the input stream for nitrogen is represented as $\%N_2$, and for analyte as $\%X$. Note that $FR_{N_2}$is set to 1 as a reference for the other analytes and is not included in the equations.

The peak area of analyte X in the chromatogram, A_X, combined with its response factor, allowed us to calculate the conversion of CO_2 (X_{CO_2}) using the following calculations:

$$X_{CO_2} = 100 * \frac{Input - Output}{Input}$$

where input refers to the CO_2 and N_2 introduced into the reactor, while output is the total amount of untransformed CO_2. Therefore, by substituting in the equation:

$$X_{CO_2} = 100 * \frac{\frac{\%CO_{2,in}}{\%N_{2,\,in}} - \frac{A_{CO_2}}{FR_{CO_2}A_{N_2}}}{\frac{\%CO_{2,in}}{\%N_{2,\,in}}}$$

where $\%CO_{2,in}$ and $\%N_{2,\,in}$ are the known percentage of CO_2 and N_2, respectively, in the input stream, A_{CO_2} is the chromatographic area of the CO_2 analysed in the output stream, A_{N_2} is the chromatographic area of the N_2 analysed in the output stream, FR_{CO_2} is the calculated response factor of the CO_2 and $\sum \%X$ is the sum of the percentages of all the analyzed species derived from the hydrogenation of CO_2.

Then, to calculate selectivity (S_X):

$$S_x = \frac{\%X_{Out}}{\sum \%X_{Out}}$$

Where $\%X_{Out}$ in the percentage of any given substance X analysed from the output.

S1.2. Kinetic experiments

For the kinetic experiments, the reaction gas flow was systematically increased from 32 mL/min to 1000 mL/min to determine the reaction rate constants under both conventional heating and magnetic induction heating. Specifically, 200 mg of the corresponding catalyst (**Co@C** and **CoPd/Co@C**) was placed in a quartz reactor positioned at the center of the AMF coil, through which different CO_2:H_2 (1:3) mixtures were introduced at flow rates ranging from 32 to 1000 mL/min. This variation consequently adjusted the GHSV from 42 to 1300 min^{-1}, respectively. The reaction rate constants under conventional heating were estimated at temperatures of 450, 500, 550, and 600 ºC (see Figure S8.2 and Table S8.4). Meanwhile, the reaction kinetics were studied for magnetic induction heating using field amplitudes of 49, 53, 57, 61, and 63 mT (see Figure S8.3 and Table S8.5). To mitigate the potential overpressure problems in the reactor caused by high flow rates, a relief valve was installed upstream of the chromatogram inlet, preventing erroneous increases in conversion measurements.

S1.3. Diffusion catalytic studies

To determine whether the magnetically induced catalytic reactor system is affected by diffusion limitations, several catalytic tests were conducted. First, the effect of particle size on the activity of the catalysts (**Co@C** and **CoPd/Co@C**) was evaluated to assess the presence of intraparticle diffusion limitations. For this purpose, both catalysts were sieved into three different particle size ranges: 0.2–0.4 µm, 0.4–0.6 µm, and 0.6–0.8 µm, and their catalytic activity was compared at 450 ºC under the same GHSV (42.1 min^{-1}). As shown in Table S1.1, no significant dependence of conversion on particle size was observed, confirming that the system does not exhibit intraparticle diffusion limitations.

Table S1.1. Comparative analysis of the particle size effect on **Co@C** and **CoPd/Co@C** to study the presence of intraparticle diffusion limitations.

Catalyst	Particle size (µm)	Conversion (%)	Selectivity (%)
**Co@C**	0.2-0.4	23.6	>99
	0.4-0.6	22.9	>99
	0.6-0.8	23.0	>99
**CoPd/Co@C**	0.2-0.4	21.5	>99
	0.4-0.6	22.1	>99
	0.6-0.8	21.3	>99

On the other hand, to verify whether the magnetically induced reactor exhibits interparticle diffusion limitations, we compared the activity of **CoPd/Co@C** by varying the amount of catalyst introduced into the quartz reactor and adjusting the gas flow rate to maintain a constant GHSV in all cases. As shown in Figure S1.1, when changing the catalyst mass between 175, 200, and 225 mg, while keeping the

GHSV constant at 132 min^{-1}, the catalytic activity remained nearly constant over six hours of reaction, with no significant evidence of interparticle diffusion limitations.

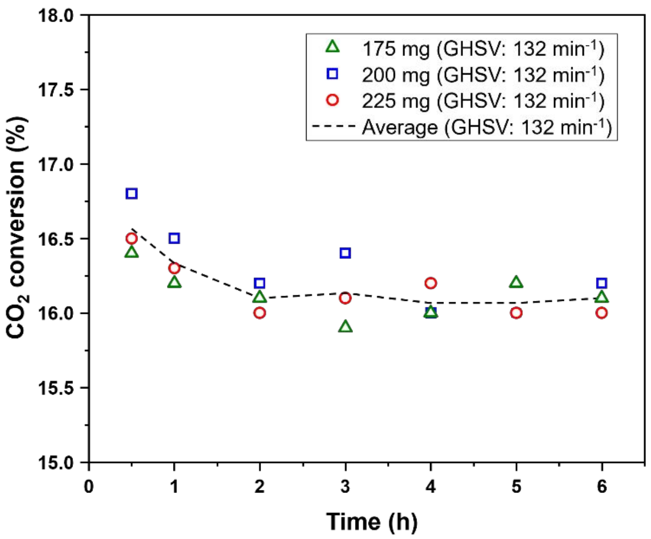

Figure S1.1. Comparative study of the activity of different amounts of **CoPd/Co@C** at the same GHSV (min^{-1}) over 6 hours of magnetically induced reaction.

S2. Experimental catalytic setup

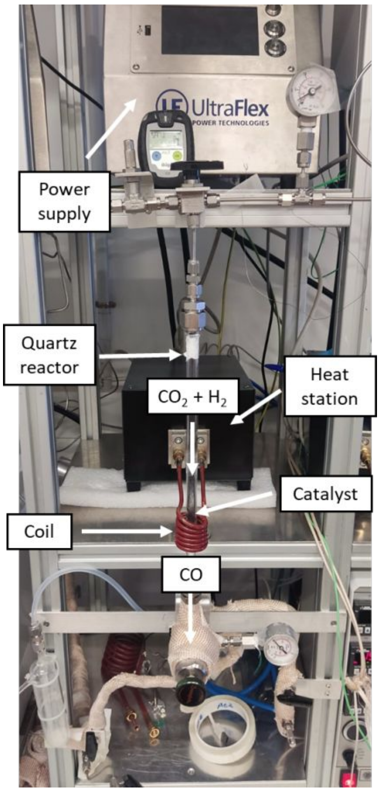

Figure S2.1. Picture of the catalytic set-up used in the magnetically induced RWGS reaction.

S3. *In situ* and *ex-situ* combined X-ray diffraction (XRD) and X-ray absorption spectroscopy (XAS).

Ex situ powder diffraction patterns of the 2D-MOF, with comparisons to the pyrolysed samples, are presented for the monometallic Co and bimetallic CoPd catalysts (Figure S3.1). The data for **Co@C** and **CoPd/Co@C** is a repeat of that shown in the main text (Figure 1), but kept at their original measured wavelength**.** For **Co@C**, the broad peak of semi-crystalline carbon at 2θ: 8º is mostly masked by a reflection of quartz as the data was acquired from powder in a capillary rather than a pressed pellet. The structural information obtained from full profile refinements is given in Table S3.1, with the fits presented in Figure S3.2.

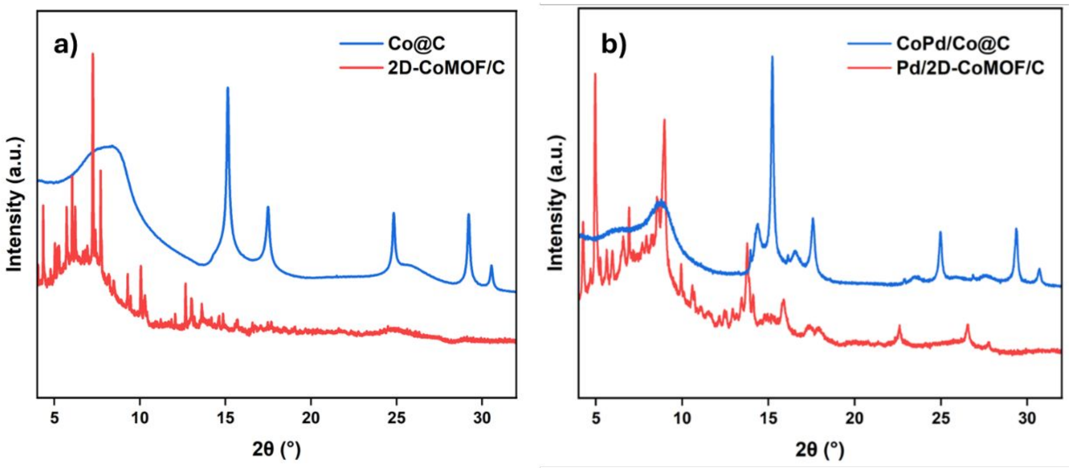

Figure S3.1. Synchrotron PXRD patterns of a) 2D-CoMOF/C and **Co@C** and b) Pd/2D-CoMOF/C and **CoPd/Co@C**, measured at ALBA synchrotron at 23keV, 2θ not converted to Cu Kα to match refined data below.

The two additional phases present in **CoPd/Co@C** compared to the monometallic catalyst, **Co@C,** are observed in Figure S3.2b. The full profile refinement confirmed these phases as *fcc*-Pd^0, and an *fcc*-CoPd alloy. The Pd^0 phase is a minor component with small, sharp reflections, presumed to be pure and contributing only 0.5 wt% to the total crystalline fraction, while the Co^0 and CoPd alloy phases contribute 72.9 and 26.6 wt%, respectively. Due to the weak intensity of the Pd^0 reflections, the crystallite size could not be confidently determined. The crystallite size sizes of the other phases are given in Table S3.1.

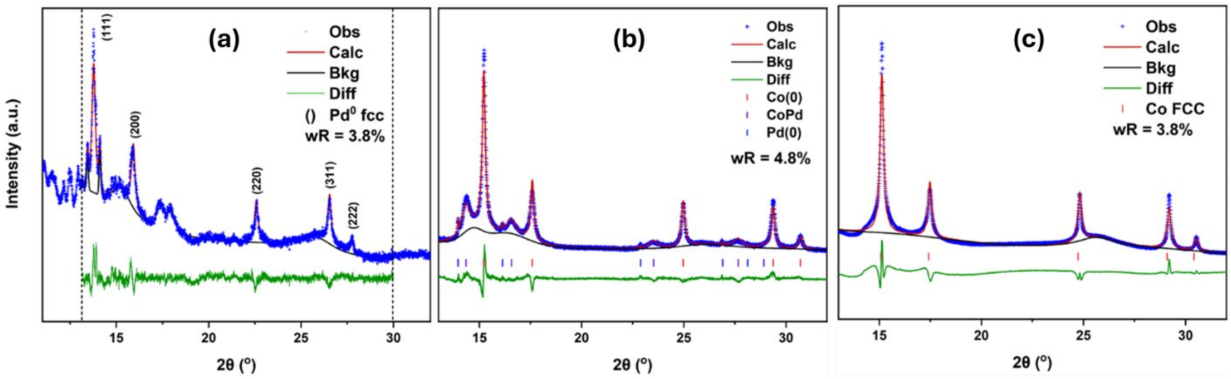

Figure S3.2. Refined fit of synchrotron PXRD of (a) Pd/2D-CoMOF/C, (b) pyrolysed **CoPd/Co@C**, and (c) **Co@C**, fit to three different metallic phases. In fit (a), reflections of the MOF were added to the background, and only the Pd^0 phase was fit to determine crystallite size, and the 2θ range refined was shortened.

Table S3.1. Rietveld refinement values of Pd/2D-CoMOF/C fit to Pd^0 (wR = 3.8%), **CoPd/Co@C** fit to three different metallic phases (wR = 4.8%), and of **Co@C** fit to Co^0 (wR = 3.8%). A march-dollarse ratio of 1.4 was applied along the (111) plane of the Co^0 phase to account for stacking faults. Parameters marked with a * were fixed during refinement.

	**Pd/2D-CoMOF/C**	**Co@C**	**CoPd/Co@C**		
**Phase**	**Pd^0**	**Co^0 fcc**	**Co^0 fcc**	**Pd^0 fcc**	**CoPd fcc**
**Lattice parameter (Å)**	3.89	3.55	3.54	3.84	3.74
**Size (nm)**	15	15	17	N/A	7
**Stoichiometry**	Pd_1	Co_1	Co_1	Pd_1	$Co_{0.33}Pd_{0.67}$
**Phase wt %**	N/A	100%	72.90%	0.50%	26.60%
**U_{iso}**	0.008	0.009	0.009	0.0084	0.01*, 0.01*

Refinement of the fractional occupancy of the alloy phase resulted in a stoichiometry of $Co_{0.33}Pd_{0.67}$, which is in agreement with a calibration curve of the observed lattice parameters of the pure *fcc*-Co and *fcc*-Pd phases, according to Vegard's law for solid solutions (Figure S3.3).

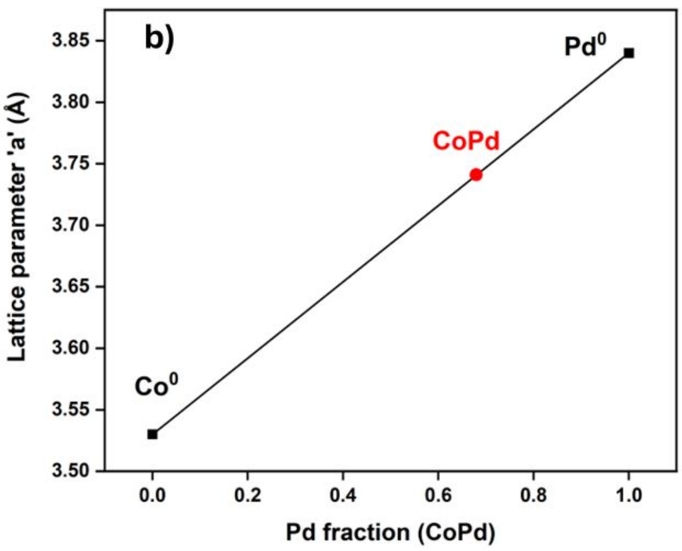

Figure S3.3. Fitting of the lattice parameter of the CoPd alloy phase to a calibration curve generated from the lattice parameters of *fcc*-Co^0 and *fcc*-Pd^0, demonstrating a resulting stoichiometry of $Co_{0.33}Pd_{0.67}$.

A more complete description of the observation of the *in situ* PXRD experiment, shown in Figure 1 in the main text, is now given:

Figure 1 in the main text shows the *in situ* PXRD and Pd K-edge spectra collected during a temperature ramp, resulting in the formation of the **CoPd/Co@C** catalyst from Pd/2D-CoMOF/C. The *in situ* PXRD reveals the decomposition of the MOF between 100–150 ºC, followed by several crystalline carbon phase transformations at higher temperatures. The Pd^0 reflections, observable from room temperature, become broader and less intense until 450 ºC, at which point they disappear completely, coinciding precisely with the appearance of the Co^0 reflections, corresponding to the formation of the Co NPs, which become more crystalline with increasing temperature. At the same temperature (450 ºC), another set of reflections emerges, corresponding to the CoPd alloy formation. Therefore, *in situ* PXRD confirms that the bimetallic Pd/2D-CoMOF/C initially consists of small Pd^0 NPs dispersed over 2D-CoMOF/C. During pyrolysis, these Pd^0 NPs disappear, leading to the formation of small CoPd alloy nanoparticles. At temperatures above 650 ºC, small reflections from a new Pd^0 phase are observed. Note that the 2θ positions of all phases also shift with temperature due to thermal expansion.

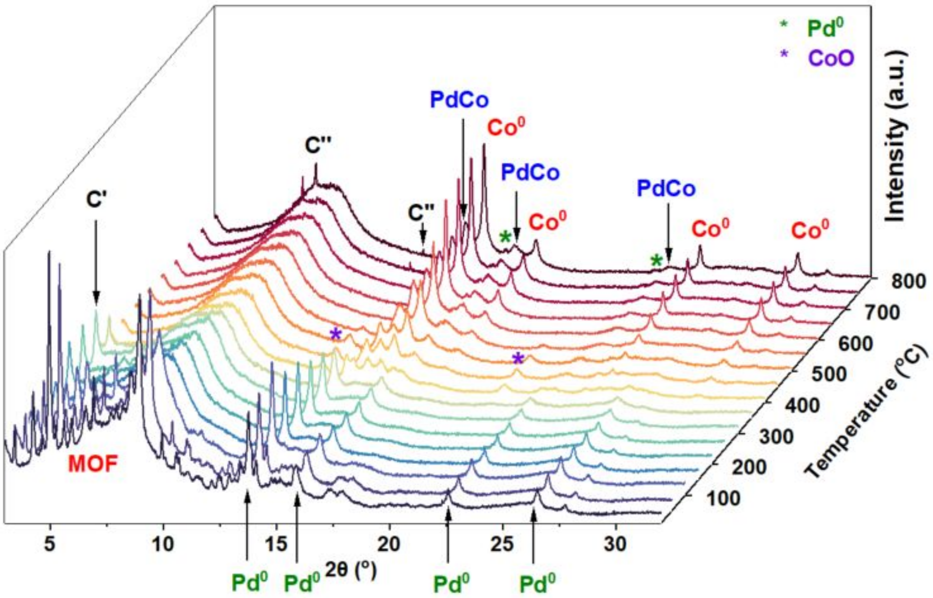

Figure S3.4. *In situ* PXRD during *in situ* pyrolysis of Pd/2D-CoMOF/C, measured at 23keV each 50°C, equivalent to Figure 1a in the main text but with a wider 2θ range and more extensive phase labelling.

The various phase transitions observed in the above in situ PXRD figure, which are not discussed in the main text (Co^0, Pd^0 and PdCo), are now described. In order to increase the temperature after decomposition of the MOF to approximately 100°C, First, a broad and intense peak emerges centred at 8.5° 2θ, which remains consistent throughout the ramp. This corresponds to the (002) reflection of turbostatic carbon,[1] resulting here from the thick carbon shell coating the nanoparticles. Being a spherical shell, these carbon layers are haphazardly arranged and rotationally randomised, hence the breadth of the reflection. A graphene oxide phase forms alongside this, labelled C', with the (002) reflection observed at 3.6° 2θ. This phase decomposes at 350-400 °C.[2] This is followed by formation of CoO (fm3m phase), at 400°C. The metallic Co phase forms at approximately the same time as this one, however, the metallic phase is permanent, whereas the oxide is lost entirely by 550°C. The final phase carbonaceous phase, labelled C'',[1] appears at temperatures above 700°C. These particularly sharp reflections at 9.1°.and 12.2° 2θ are the (002) and (004) reflections of graphite.[2]

The transition from Pd^0 to a CoPd alloyed phase is also observed in the χ(k) data from *in situ* XAFS analysis, collected concurrently with PXRD analysis (Figure 1c, main text). As thermal disorder increases, all oscillations dampen, and notably, the positive peak at 5.8 $Å^{-1}$ is partially lost at 400 ºC and completely disappears by 500 ºC. This onset of alloying by XAFS at 400 ºC occurs slightly earlier than observed by PXRD, likely due to the delay necessary for the new phase to become crystalline. The loss of Pd^0 occurring with the formation of CoPd and the lack of shifting of the Co^0 phase once present suggests that the alloy forms by Co insertion into the Pd phase. This is consistent with the Co atoms,

initially dispersed in the 2D-CoMOF, migrating to aggregate and form NPs at the same temperature at which the alloy forms.

The XANES (Figure S3.5a) and fourier transform magnitude (Figure S3.5b) of the sample pre and post pyrolysis (Pd/2C-CoMOF/C and CoPd/C@C respectively), *i.e.* from the start and end of the *in situ* pyrolysis but collected at room temperature, are shown, in particular highlighting the addition of a destructive scattering path at 24250 eV in the XANES and the corresponding new Pd-Co path at 2.1Å. The results from EXAFS fitting of these paths are presented in a later section.

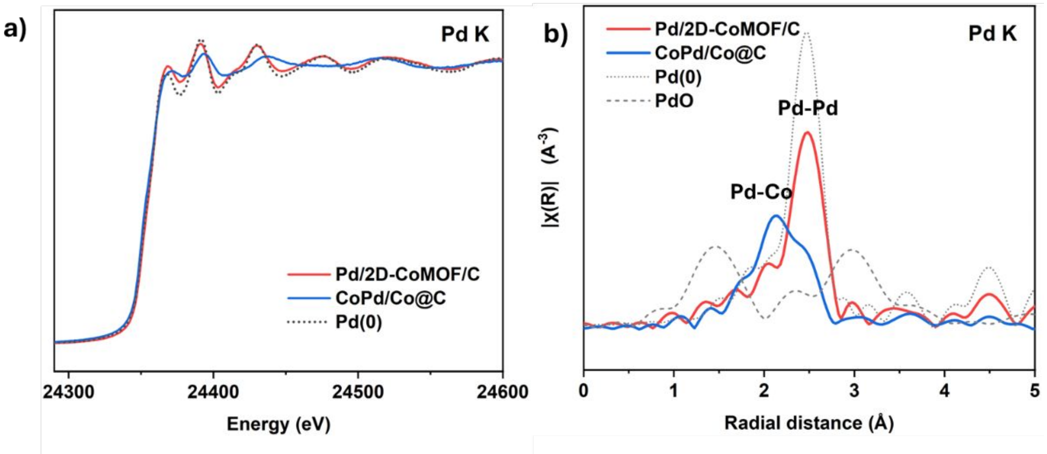

Figure S3.5. Pd K-edge XAS spectra of the Pd/2D-CoMOF/C and the pyrolysed **CoPd/Co@C**: a) normalised absorption and b) the k^2-weighted Fourier transform magnitudes. The Pd^0 reference is included for comparison.

The Co K-edge was also measured for both the monometallic and bimetallic samples before pyrolysis, and after cooling to room temperature following *in situ* pyrolysis (Figure S3.6). The monometallic **Co@C** catalyst is identical to the **CoPd/Co@C** catalyst; cobalt is fully reduced to Co^0 during the pyrolysis process. For **CoPd/Co@C**, unlike at the Pd K edge there are no new scattering paths to elements other than Co (such as O or Pd) in the Fourier transform. This is because alloying is best observed at the edge of the element of the lowest concentration; here the Co:Pd ratio is roughly 5:1 While no new scattering paths are easily identified however, there is a noticeable shift in the position in energy of the first two oscillations in the EXAFS in the normalised spectra. The position of the whiteline is unchanged, highlighting that these are changes to the coordination environment of the scattering atom which occur without any change in oxidation state, consistent with the formation of a metallic alloy. This is observed more clearly in Figure S4.5 as it does not contain overlapping metal reference spectra.

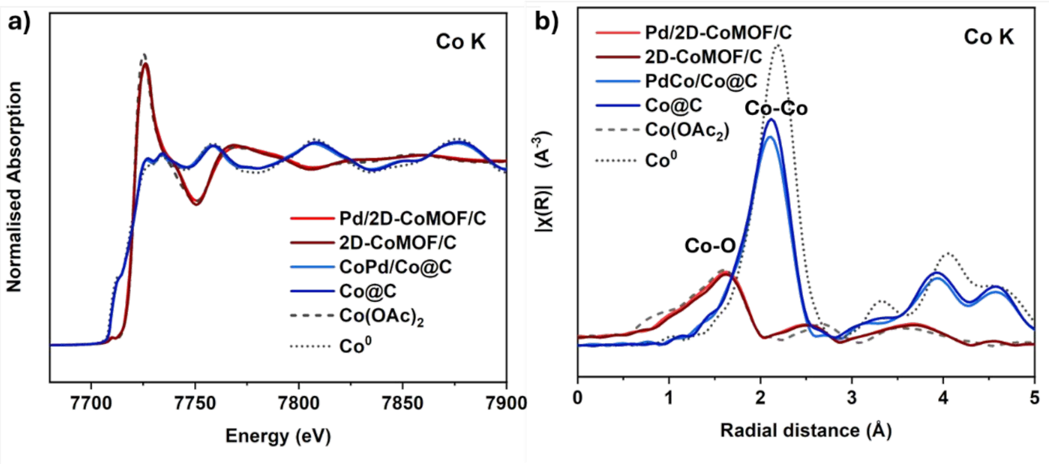

Figure S3.6. Co K-edge XAS spectra of the monometallic 2D-CoMOF/C and **Co@C**, and the bimetallic Pd/2D-CoMOF/C and **CoPd/Co@C**: (a) normalised absorption and (b) the k^2-weighted Fourier transform magnitudes. The Co^0 and a complexed Co(II), $Co(OAc)_2$, references are included for comparison.

Co K-edge and Pd K-edge data were also collected for samples after magnetic induction catalysis, displayed in Figure S3.7, with the pyrolysed sample spectra repeated for comparison. At the Co K edge, both catalysts exhibited a slight increase in the white line intensity in the XANES region, indicating partial oxidation of Co^0 during catalysis. The EXAFS remain predominantly that of Co^0, with only marginal intensity from a Co-O path which could not be confidently fit. Linear combination fitting of the XANES spectra, performed before and after the reaction using references for metallic and oxidized Co, revealed a 15 % increase in oxidized species for the monometallic catalyst, compared to only a 6% increase for the bimetallic catalyst. These results are given in Table S3.2 (*vide infra*).

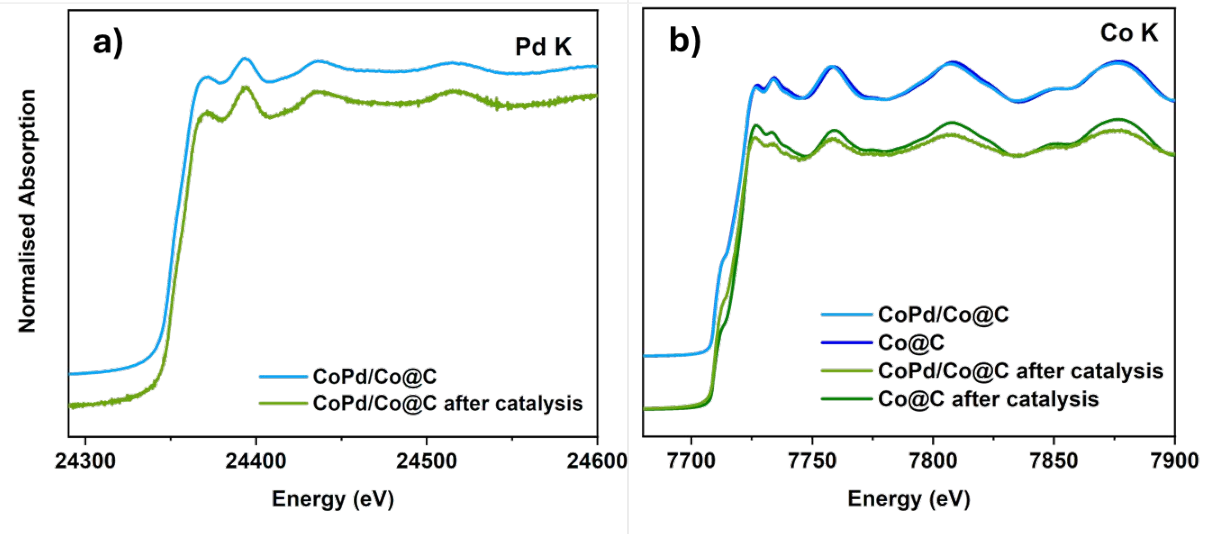

Figure S3.7. XANES at a) the Pd K edge and b) the Co K edge of **Co@C** and **CoPd/Co@C**, pyrolysed and after catalysis. The **CoPd/Co@C** catalyst was measured on B18 at DLS which was experiencing issues with monochromator stability, hence the spectral noise. The rest were measured on NOTOS at ALBA. This is likely the source of the amplitude differences in the samples after catalysis at the Co K edge.

Table S3.2. Linear combination fitting (LCF) results of **Co@C** and **CoPd/Co@C**, as-synthesized and after catalysis, fit to Co^0, CoO and Co_3O_4. The two oxidised components are summed due to the difficulty in distinguishing the two by LCF.

Catalyst	Co^0 (%)	CoO + Co_3O_4 (%)	R-factor
**Co@C**	81 ± 2.8	19 ± 3.2	0.006
**Co@C after catalysis**	66 ± 2.5	34 ± 2.7	0.004
**CoPd/Co@C**	82 ± 2.7	18 ± 3.2	0.005
**CoPd/Co@C after catalysis**	76 ± 4.1	24 ± 2.4	0.017

First-shell fits of the EXAFS for the mono- and bimetallic systems, before pyrolysis, after pyrolysis and after magnetic induction reaction were performed at both the Pd K and Co K edge. Due to the relatively large size of the formed metallic particles, fitting the first coordination shell was considered sufficient to determine the average local coordination environment. Plots of the fits are presented in Figure S3.8. The most relevant fitted parameters are displayed in Table S3.3, with full structural parameters in Table S3.4 below.

Table S3.3. Coordination number and interatomic distances from EXAFS fitting parameters for **Co@C** (Co K edge) and **CoPd/Co@C** (Co K- and Pd K edge), fitting the first coordination shell only.

	Path (abs-sc)	C.N.	R (Å)
**Co@C**	Co-Co (Co^0)	10.2 ± 0.2	2.49 ± 0.001
**CoPd/Co@C**	Co-Co (Co^0)	9.6 ± 0.4	2.49 ± 0.001
	Pd-Co (PdCo)	3.8 ± 0.1	2.58 ± 0.003
	Pd-Pd (PdCo)	4.5 ± 0.2	2.70 ± 0.004

S_0^2 = 0.86 for the Pd K edge and 0.74 for the Co K edge, as determined by corresponding metal foils. Fit range $3<k<13.2$, $1.2<R<3.1$ for Pd K edge, and $3<k<13.7$, $1.2<R<3.1$ for Co K edge. Various absorber – scatterer (abs – sc) paths are used for the fits.

The coordination numbers and bond distances of the Co component in the monometallic system (**Co@C**), were very similar to those of the Co component in the bimetallic system and in agreement with the values expected for the *fcc*-Co^0 phase. To fit the pyrolyzed bimetallic system, FEFF calculations were carried out on a *fcc*-Pd^0 structure, with 50 % of Pd atoms substituted with Co. The calculated scattering distance for the Pd-Co path is shorter than that for Pd-Pd (2.58 *vs.* 2.70 Å), which is reasonable

given the smaller atomic radius of Co compared to Pd. This aligns well with the smaller lattice size of the CoPd alloyed phase compared to the Pd^0 phase from XRD analysis (Table S3.1).

After catalysis, for the **CoPd/Co@C** catalyst, coordination numbers of the alloyed phase at the Pd K edge increase, and decrease for the metallic Co phase at the Co K-edge. This potentially indicates migration of more metallic Co into the CoPd alloy, resulting in increased sizes of the CoPd crystallites located towards the external area of the Co metal NP cores.

EXAFS fits and obtained structural parameters for all discussed spectra:

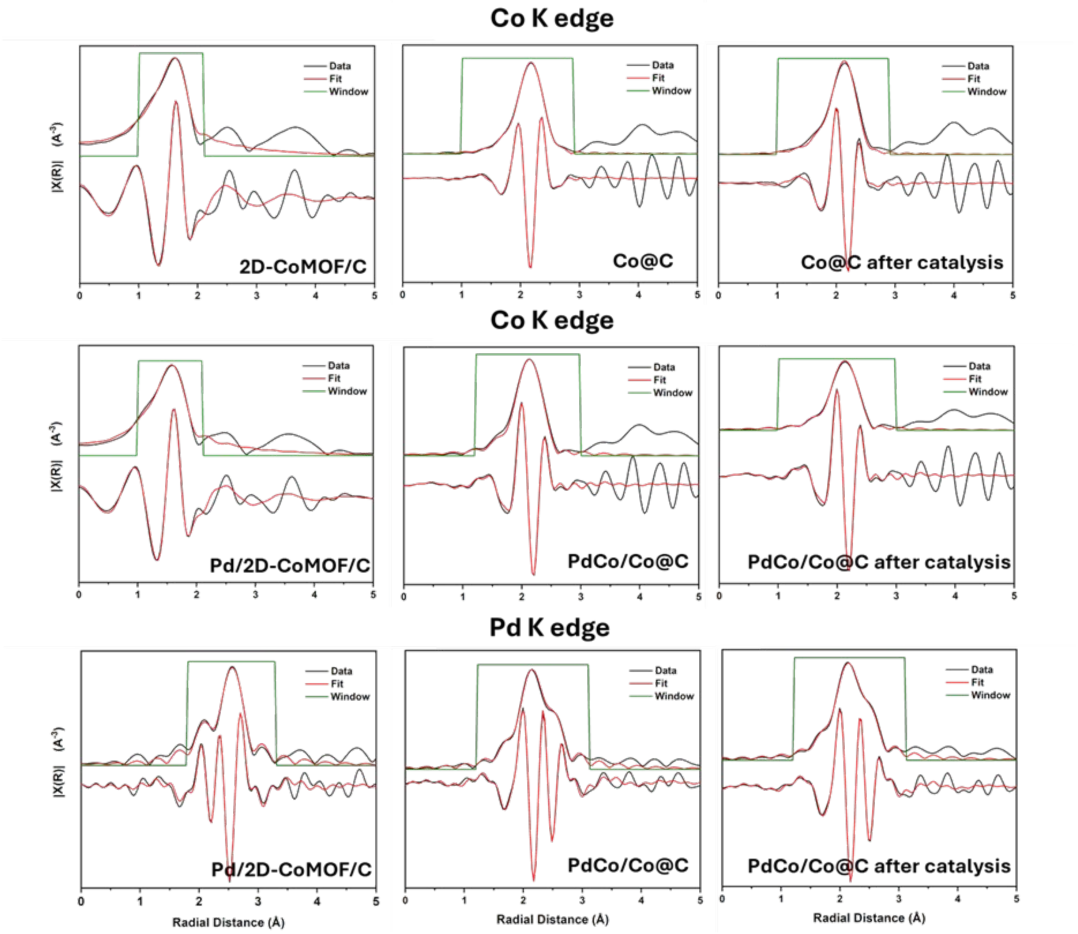

Figure S3.8. Co K and Pd K-edge k^2 weighted EXAFS spectra, real and magnitude, including fit and fitting window, for all *ex-situ* samples measured at either NOTOS at ALBA synchrotron (MOF, pyrolyzed) or B18 at the Diamond light source (post-reaction).

Tables S3.4. EXAFS data obtained from **Co@C** and **CoPd/Co@C** as-synthesized and after magnetic induced catalysis. Pyrolysed samples were measured on NOTOS beamline at ALBA. Post-reaction samples were measured on B18 at the Diamond Light source as part of the UK Catalysis Hub Block Allocation Group Access. The amplitude ($S0^2$) was refined from foils of the relevant metal from the relevant beamline for each sample.

Pd K edge: *Pellets measured at ALBA used amp value of 0.861. Pellet labelled * measured at Diamond, amp value of 0.827. Data were fit in the range R 1.2-3.1 with a K window 3-13.2.*

Material	E0 (eV)	Path (abs-sc)	C.N.	σ^2 x10^{-3}	R (Å)	R_{fac}
**Pd/2D-CoMOF/C**	4.1 ± 0.5	Pd-Pd (Pd^0)	9.6 ± 0.6	6.2 ± 0.4	2.75 ± 0.003	0.003
**CoPd/Co@C**	2.3 ± 0.4	Pd-Pd (PdCo)	3.5 ± 0.1	6.1 ± 0.3	2.70 ± 0.002	0.004
		Pd-Co (PdCo)	3.8 ± 0.1	6.8 ± 0.3	2.58 ± 0.003	
**CoPd/Co@C after catalysis***	-0.2 ± 0.2	Pd-Pd (PdCo)	4.5 ± 0.2	6.6 ± 0.2	2.70 ± 0.004	0.003
		Pd-Co (PdCo)	5.0 ± 0.1	6.7 ± 0.2	2.57 ± 0.003	

Co K edge: *Pellets measured at ALBA used amp value of 0.741. Pellet labelled * measured at Diamond, amp value of 0.612. The sample containing only CoO paths was only fit in the range R 1-2.1, K window 3-11.5. Others were fit in the range R 1.2-3.1, K window.*

Material	E0 (eV)	Path (abs-sc)	C.N.	σ^2 x10^{-3}	R (Å)	R_{fac}
**Pd/2D-CoMOF/C**	-4.2 ± 0.3	Co-O (CoO)	6.0 ± 0.5	8.4 ± 1.0	2.06 ± 0.003	0.005
**CoPd/Co@C**	-0.3 ± 0.3	Co-Co (Co^0)	9.6 ± 0.4	6.4 ± 0.2	2.49 ± 0.001	0.005
**CoPd/Co@C After catalysis***	-0.5 ± 0.4	Co-Co (Co^0)	6.9 ± 0.3	5.5 ± 0.3	2.49 ± 0.002	0.009
**2D-CoMOF/C**	-3.7 ± 0.9	Co-O (CoO)	6.5 ± 0.2	8.4 ± 0.1	2.08 ± 0.01	0.008
**Co@C**	7.3 ± 0.1	Co-Co (Co^0)	10.2 ± 0.2	6.7 ± 0.1	2.49 ± 0.001	0.001
**Co@C after catalysis***	7.3 ± 0.1	Co-Co (Co^0)	8.6 ± 0.6	6.5 ± 0.3	2.49 ± 0.000	0.011

S4. Field emission scanning electron microscopy (FESEM)

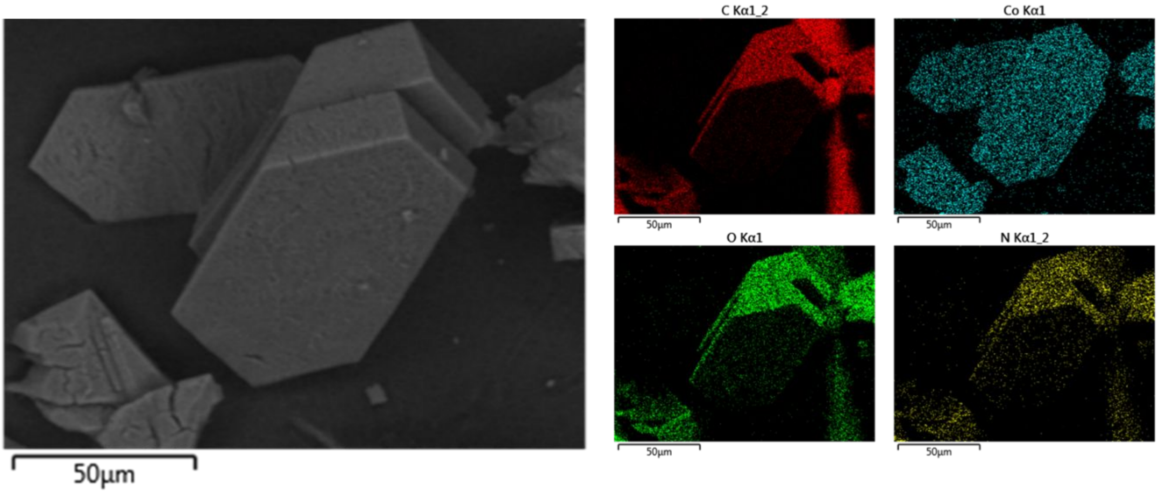

Figure S4.1. FESEM image coupled with EDX analysis of 2D-CoMOF.

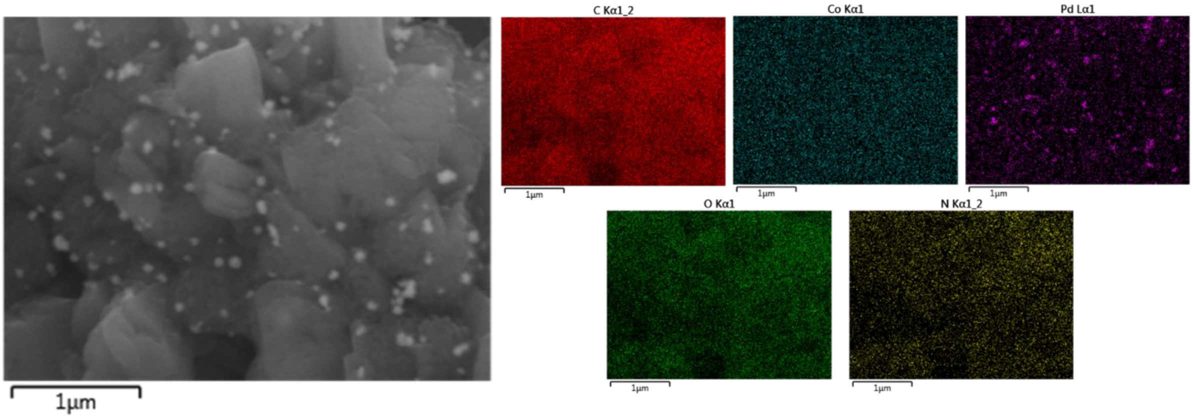

Figure S4.2. FESEM image coupled with EDX analysis of Pd/2D-CoMOF.

S5. XPS

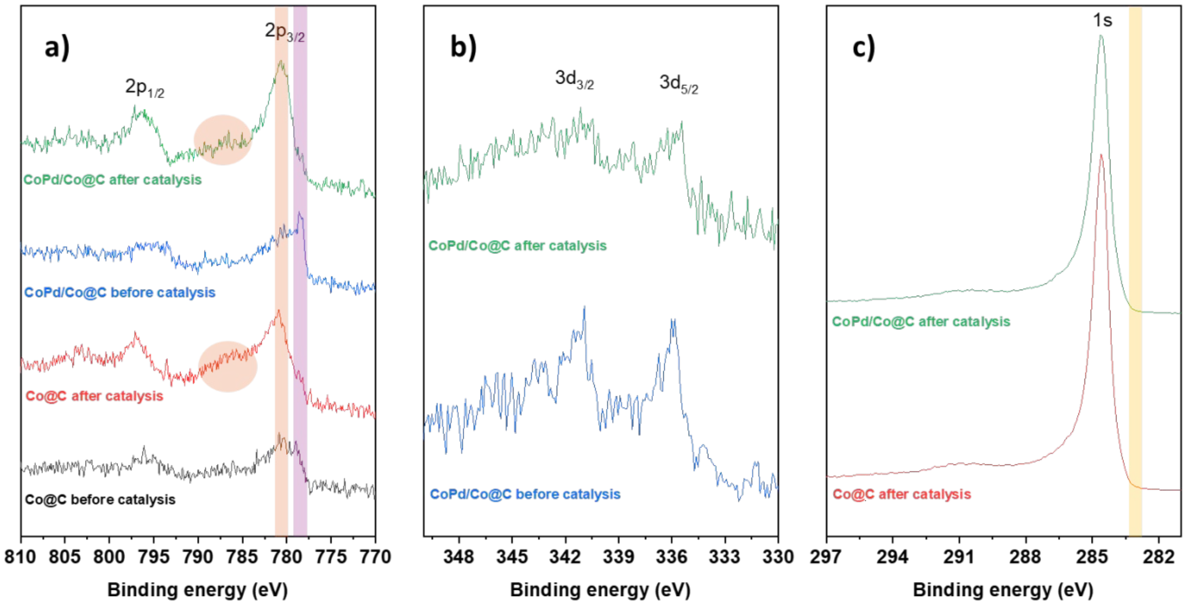

Figure S5.1. Comparison of the XPS spectra of the monometallic catalysts, **Co@C**, before (black) and after (red) catalytic tests, along with their bimetallic analogues, **CoPd/Co@C**, before (blue) and after (green) catalysis, in the regions of (a) Co 2p and (b) Pd 3d. For comparison purposes, in panel (a), the positions corresponding to the metallic state of cobalt (Co^0) (778 eV) are marked in pink, and those for cobalt oxides (780–781 eV) are marked in orange. The characteristic satellite structure of cobalt oxides (785-790 eV) is also indicated in orange. In panel (c), the position corresponding to the carbide (~283 eV) is marked in yellow.

S6. RAMAN

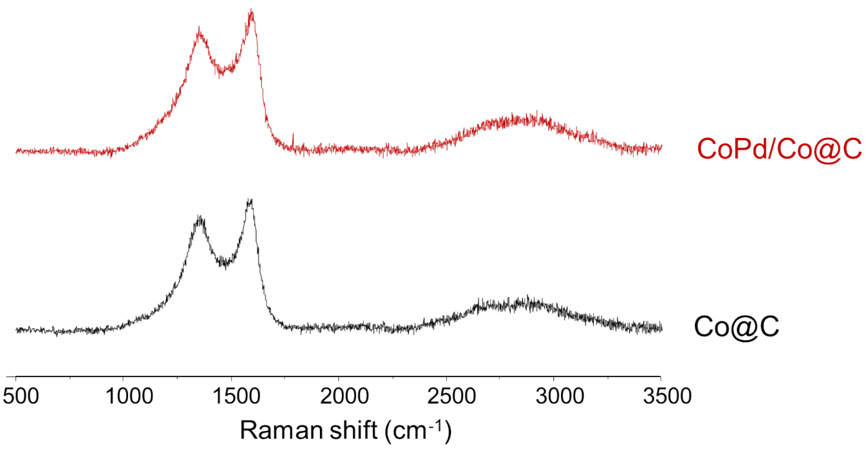

Figure S6.1. Raman spectra of **Co@C** and **CoPd/Co@C**.

S7. SAR measurements

SAR has been measured by calorimetry. Approximately 30 mg of the heating agents (**Co@C** and **CoPd/Co@C**) were introduced to a quartz tube, which was then placed in a calorimeter containing 1.8 mL of deionized water. The temperature was monitored throughout the experiment. The sample was exposed to different alternating magnetic fields (0-47 mT), oscillating with a frequency of 93 kHz for 50 seconds. At the end of the magnetic field application, the temperature rise was measured after shaking the calorimeter to ensure temperature homogeneity. This temperature was recorded using two probes positioned at the top and bottom of the calorimeter. The temperature rise was determined from the mean slope of the ΔT/Δt function after this process. The raw SAR values were then calculated using the following expression:

$$SAR = \frac{\sum_i cp_i \cdot m_i}{m_{metal}} \cdot \frac{\Delta T}{\Delta t}$$

where Cp_i and m_i are the specific heat capacity and the mass for each component respectively (Cp_{Co} = 421 $J \cdot kg^{-1} \cdot K^{-1}$, Cp_{Pd} = 240 $J \cdot kg^{-1} \cdot K^{-1}$, Cp = 4186 $J \cdot kg^{-1} \cdot K^{-1}$ for water and Cp = 720 $J \cdot kg^{-1} \cdot K^{-1}$ for glass), and m_{metal} is the mass of the pure metal in the sample.

Table S7.1. Values of M_S, $M_{R,}$ and SAR of **Co@C** and **CoPd/Co@C**, as synthesized and after catalysis.

**Catalyst**	**M_S ($A \cdot m^2 \cdot kg_{Co}^{-1}$)**		**M_R ($A \cdot m^2 \cdot kg_{Co}^{-1}$)**		**SAR ($W \cdot g^{-1}$)**
	**5 K**	**300 K**	**5 K**	**300K**	
**Co@C**	172	165	74	44	144
**Co@C after catalysis**	139	132	52	26	68
**CoPd/Co@C**	137	135	57	44	69
**CoPd/Co@C after catalysis**	105	95	46	23	62

S8. Magnetically induced catalytic results

Table S8.1. Values of temperature, conversion, and selectivity for magnetic induced catalysis with **Co@C** and **CoPd/Co@C** catalysts and theoretical equilibrium CO_2 conversion.

Catalyst	MF (%)	MF (mT)	T (°C)	X CO_2 (%)	S CH_4 (%)	S CO (%)	X CO_2 Eq (%)
**Co@C**	30	35	196	1.5	0.0	100.0	10.1
	40	40	254	3.9	0.3	99.7	16.8
	50	45	298	9.8	0.5	99.5	22.6
	60	49	354	28.4	0.6	99.4	30.5
	70	53	392	44.8	0.5	99.5	35.8
	80	57	423	52.8	0.4	99.6	40.0
	90	61	451	59.0	0.3	99.7	43.7
	100	63	476	61.1	0.3	99.7	46.8
**CoPd/Co@C**	30	35	192	1.9	0.4	99.6	9.7
	40	40	243	10.2	1.0	99.0	15.4
	50	45	292	41.7	0.3	99.7	21.8
	60	49	341	57.2	0.4	99.6	28.6
	70	53	379	61.4	0.4	99.6	34.0
	80	57	417	64.9	0.4	99.6	39.2
	90	61	446	67.8	0.3	99.7	43.0
	100	63	471	71.1	0.1	99.9	46.2

Table S8.2. Values of temperature, conversion, and selectivity for conventional heating catalysis with **Co@C** and **CoPd/Co@C** catalysts and theoretical equilibrium CO_2 conversion.

Catalyst	T (°C)	X CO_2 (%)	S CH_4 (%)	S CO (%)	X CO_2 Eq (%)
**Co@C**	200	0.0	0.0	100.0	10.4
	250	0.2	0.0	100.0	16.2
	300	2.8	0.2	99.8	22.8
	350	6.8	1.0	99.0	29.7
	400	15.6	1.7	98.3	36.7
	450	23.6	1.8	98.2	43.3
	500	31.1	1.7	98.3	49.4
	550	36.7	1.4	98.6	55.2
	600	42.1	0.7	99.3	60.0
**CoPd/Co@C**	200	0.8	1.8	98.2	10.4
	250	1.4	1.6	98.4	16.2
	300	1.5	1.4	98.6	22.8
	350	3.3	1.4	98.6	29.7
	400	8.8	0.9	99.1	36.7
	450	21.5	0.3	99.7	43.3
	500	33.4	0.0	99.9	49.4
	550	42.3	0.0	99.9	55.2
	600	51.5	0.1	99.9	60.0

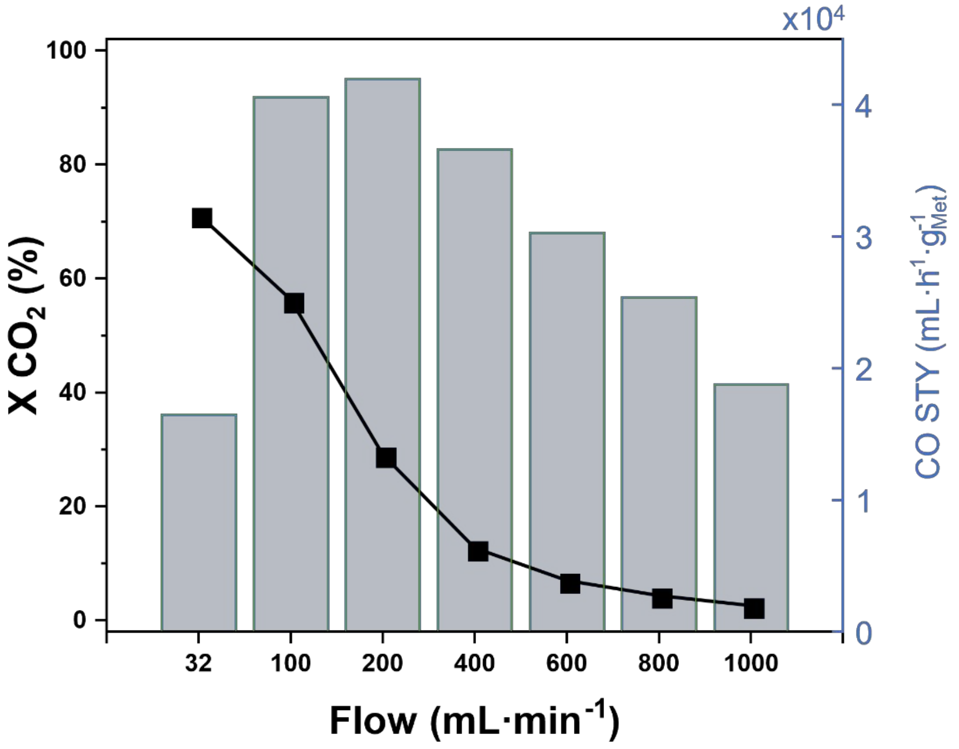

Figure S8.1. Magnetically induced RWGS with **CoPd/Co@C**, representing the Space-Time-Yield (STY) of CO and the conversion at each flow.

Table S8.3. RWGS for conventionally heated benchmark catalysts for the same space velocity as **Co@C** and **CoPd/Co@C**.

Catalyst	T (ºC)	X CO_2 (%)	S CH_4 (%)	S CO (%)	X CO_2 Eq (%)
**CZA**	200	0.1	0.0	100.0	10.4
	250	0.7	0.2	99.8	16.2
	300	3.0	0.3	99.7	22.8
	350	8.7	0.1	99.9	29.7
	400	16.0	0.2	99.8	36.7
	450	26.7	0.2	99.8	43.3
	500	37.4	0.4	99.6	49.4
	550	47.5	0.4	99.6	55.2
	600	54.7	0.3	99.7	60.0
**Pd/C** (10 wt%)	200	0.0	0.0	0.0	10.4
	250	0.0	0.0	0.0	16.2
	300	0.4	0.1	99.9	22.8
	350	2.1	0.1	99.9	29.7
	400	9.9	0.2	99.8	36.7
	450	22.3	2.3	97.7	43.3
	500	38.5	6.2	93.8	49.4
	550	47.8	14.9	85.1	55.2
	600	55.9	25.3	74.1	60.0

Table S8.4. Values of conversion at different temperatures and flows for **Co@C** and **CoPd/Co@C** under conventional heating for Ea calculations.

Catalyst	Flow (mL/min)	GHSV (min^{-1})	X CO_2 (%)			
			450ºC	500ºC	550ºC	600ºC
**Co@C**	32	42.1	23.6	31.1	36.7	42.1
	100	131.6	9.2	15.7	22.5	30.1
	200	263.2	6.0	11.0	16.2	24.7
	400	526.3	3.7	7.8	13.2	21.8
	600	789.5	3.1	6.6	11.9	20.0
	800	1052.6	2.5	6.0	10.9	18.5
	1000	1315.8	2.1	5.5	10.1	17.7
**CoPd/Co@C**	32	42.1	21.5	33.4	44.3	53.5
	100	131.6	12.0	22.8	35.2	47.5
	200	263.2	9.0	17.8	28.5	42.4
	400	526.3	6.1	13.8	23.7	37.0
	600	789.5	5.1	12.1	22.0	35.0
	800	1052.6	4.5	11.4	20.5	33.0
	1000	1315.8	3.5	10.6	19.9	32.3

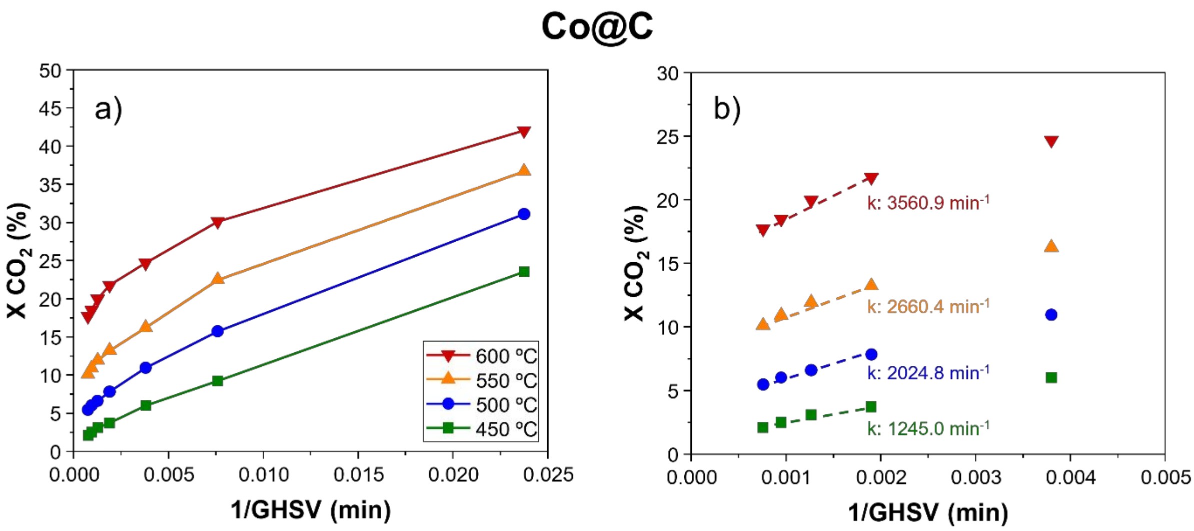

c)

k (min^{-1})	ln k	T (K)	$10^3/T$ (K^{-1})
1245.0	7.1	723	1.4
2024.8	7.6	773	1.3
2660.4	7.9	823	1.2
3560.9	8.2	873	1.1

Figure S8.2. a) Kinetics and b) plots with conventionally heated catalysis of **Co@C** for Arrhenius calculations at different temperatures and flows; and c) data used in Ea calculations extracted from Arrhenius plots.

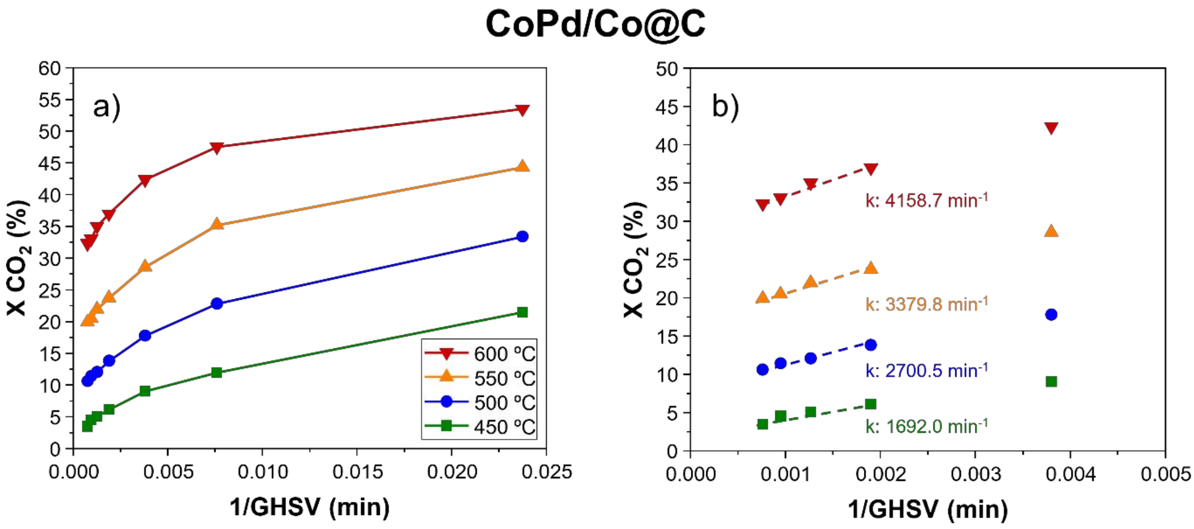

c)

k (min^{-1})	ln k	T (K)	10^3/T (K^{-1})
1692.0	7.4	723	1.4
2700.5	7.9	773	1.3
3379.8	8.1	823	1.2
4158.7	8.3	873	1.1

Figure S8.3. a) Kinetics and b) plots with conventionally heated catalysis of **CoPd/Co@C** for Arrhenius calculations at different temperatures and flows; and c) data used in Ea calculations extracted from Arrhenius plots.

Table S8.5. Values of conversion at different temperatures and flows for **Co@C** and **CoPd/Co@C** magnetically induced for RWGS reaction.

Catalyst	Flow (mL/min)	GHSV (min^{-1})	X CO_2 (%)				
			49 mT	53 mT	57 mT	61 mT	63 mT
**Co@C**	32	42.1	28.4	44.8	52.8	59.0	61.1
	100	131.6	15.7	27.7	36.1	41.2	45.2
	200	263.2	9.9	20.0	27.5	32.3	37.7
	400	526.3	5.0	14.1	21.3	24.9	27.3
	600	789.5	2.6	8.5	12.9	15.5	17.0
	800	1052.6	0.1	2.0	5.9	8.9	9.8
	1000	1315.8	-	0.1	0.0	1.8	2.0
**CoPd/Co@C**	32	42.1	57.2	61.4	64.9	67.8	71.1
	100	131.6	19.8	32.9	42.3	50.8	56.3
	200	263.2	8.9	12.3	17.7	24.5	29.1
	400	526.3	1.4	4.4	7.1	10.4	12.7
	600	789.5	0.1	1.2	3.2	5.4	7.0
	800	1052.6	-	0.1	1.2	2.7	4.4
	1000	1315.8	-	-	0.0	1.2	2.6

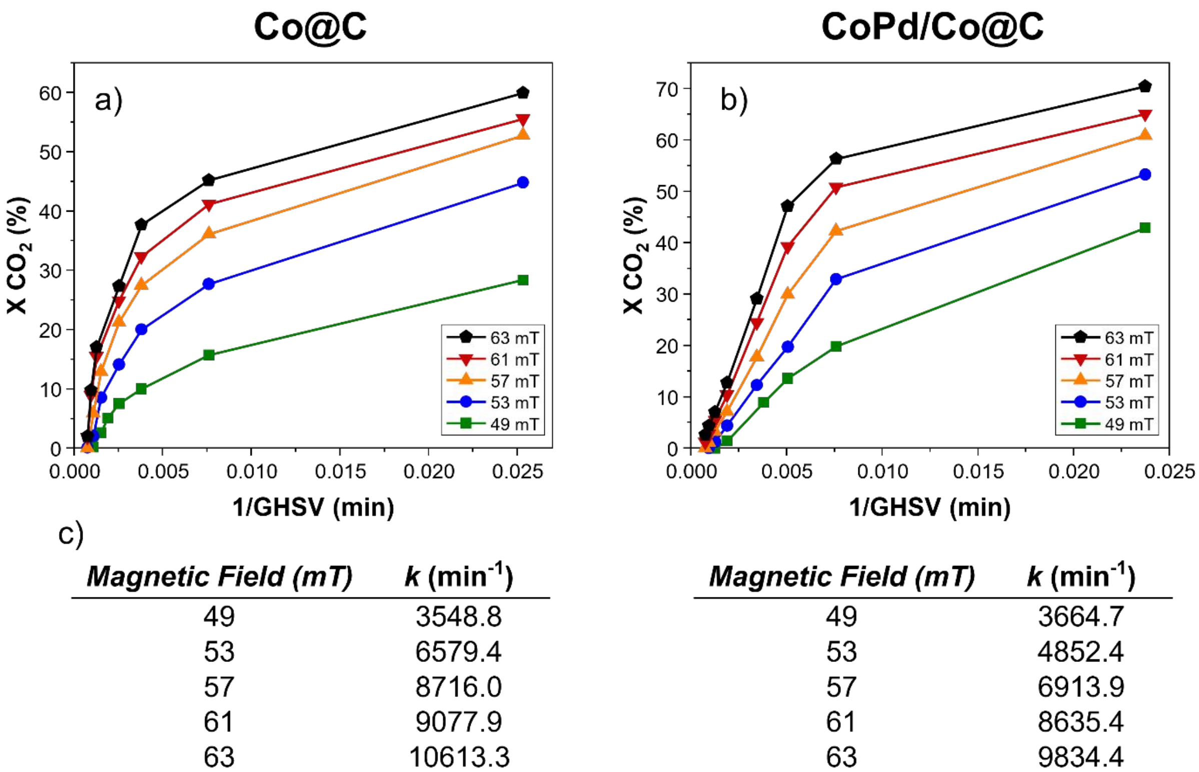

c)

Magnetic Field (mT)	***k*** **(min^{-1})**
49	3548.8
53	6579.4
57	8716.0
61	9077.9
63	10613.3

Magnetic Field (mT)	***k*** **(min^{-1})**
49	3664.7
53	4852.4
57	6913.9
61	8635.4
63	9834.4

Figure S8.4. Magnetically induced kinetics of **Co@C** (left) and **CoPd/Co@C**.

S9. State-of-the-art catalysts for CO_2 reduction

Table S9.1. Catalytic performance comparison of Co and Pd-based catalysts on RWGS.

Entry	Catalyst	Metal Loading (%)		Heating method	T (ºC)	P (bar)	H_2:CO_2 ratio	X CO_2 (%)	S CO (%)	GHSV ($mL·h^{-1}·g_{met}^{-1}$)	CO STY ($mL·h^{-1}·g_{met}^{-1}$)	CO efficiency (mL CO/kW·h)	Ref.
		Co	Pd										
1	Co/SiO_2	1.6	-	Conventional	230	25	3:1	7.5	50	87805	823	-	3
2	Na-Co/SiO_2	15	-	Conventional	380	1	3:1	32	66	300000	13306	-	4
3	Co-C-N-700	43	-	Conventional	450	1	2:1	32	90	697600	66977	-	5
5	Co/CeO_2	43	-	Conventional	340	1	4:1	35	63	69867	3081	-	6
6	Co−N−C	5	-	Conventional	500	1	4:1	52	>99	108000	11120	-	7
7	Co/SBA-15	10	-	Conventional	700	1	3:1	49	95	906641	105510	-	8
8	Pd-Co/SBA-15	10	1	Conventional	700	1	3:1	53	95	789474	99375	-	8
9	Co-CNT	5	-	Conventional	300	1	4:1	19	64	17544	427	-	9
10	Co-K-Pd/SiO_2	15	1	Conventional	270	1	3:1	19	32	5750	87	-	10
11	Co-K-Pd/SiO_2	15	3	Conventional	270	1	3:1	17	40	5111	87	-	10
12	Pd/Al_2O_3	-	0.5	Conventional	500	1	3:1	42	63	14400	953	-	11
13	Co@C	10	-	Magnetic (8 kW)	635	1	4:1	44	>99	37129	3235	16.5	12
14	Co@Ni@C	5	- (5 Ni)	Magnetic (8 kW)	698	1	4:1	74	>99	36765	5387	27.8	12
15	3%Ru/Fe	-	-	Magnetic (not specified)	481	1	4:1	41	>99	1500	123	-	13
16	Co@C[c]	11.1	-	Magnetic (2 kW)	476	1	3:1	61	>99	93200	14071	146.4	This work
17	CoPd/Co@C[c]	10.3	2.2	Magnetic (2 kW)	471	1	3:1	71	>99	93200	16378	170.4	This work
18	CoPd/Co@C[d]	10.3	2.2	Magnetic (2 kW)	369	1	3:1	29	>99	640777	46317	478.5	This work
19	CZA	-	100 (Cu, Zn, Al)	Conventional	600	1	3:1	55	>99	93200	12707	-	Standard
20	Pd/C	-	10	Conventional	600	1	3:1	56	74	93200	9652	-	Standard

[a]GHSV = gas hourly space velocity (gas flow rate/catalyst mass). [b]STY = Space-Time-Yield. [c]Flow rate of 32 $mL·min^{-1}$ CO_2:H_2 (1:3). [d]Flow rate of 200 $mL·min^{-1}$ CO_2:H.(1:3).

S10. Absorption studies

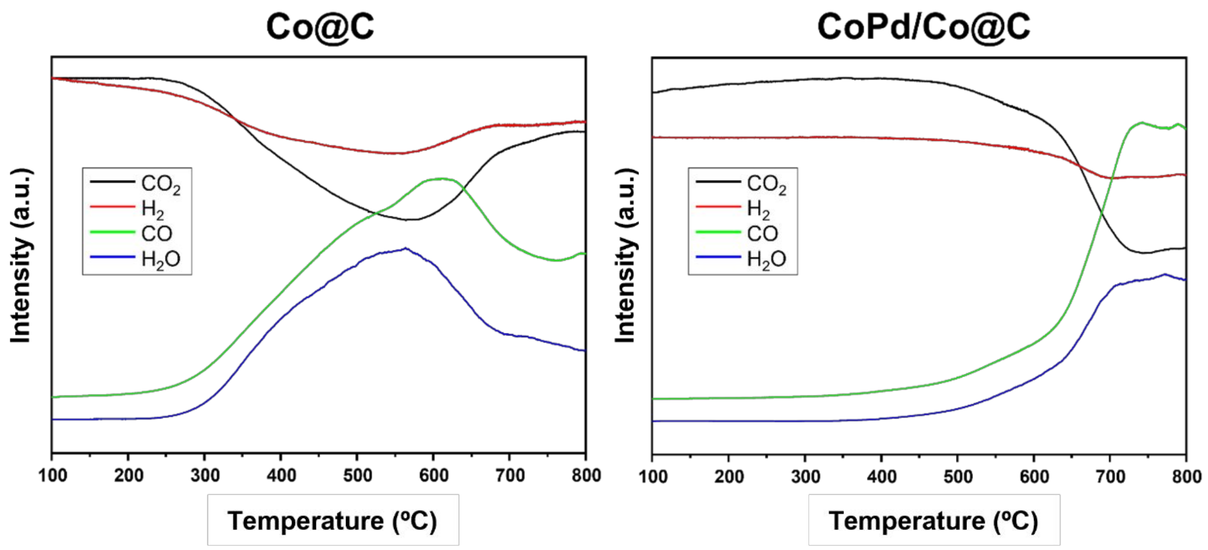

Figure S10.1. CO_2-TPR profiles of **Co@C** (*left*) and **CoPd/Co@C** (*right*), reproducing reaction conditions (CO_2:H_2 1:3).

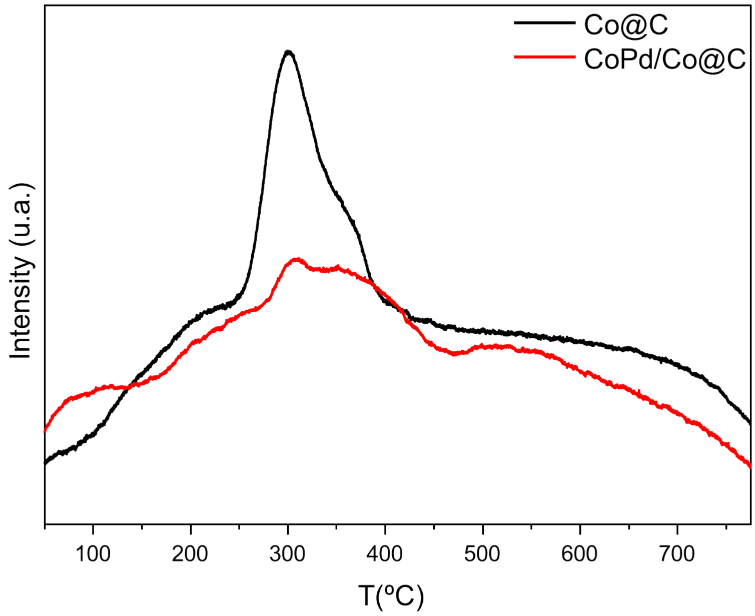

Figure S10.2. TPR analysis for **Co@C** (black) and **CoPd/Co@C** (red) after magnetic induced catalysis.

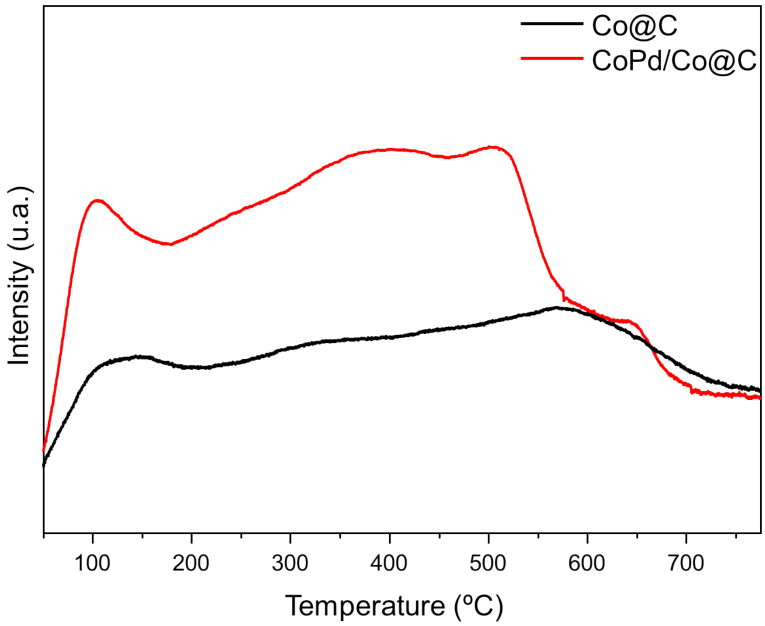

Figure S10.3. CO_2-TPD experiments for **Co@C** (black) and **CoPd/Co@C** (red) catalysts.

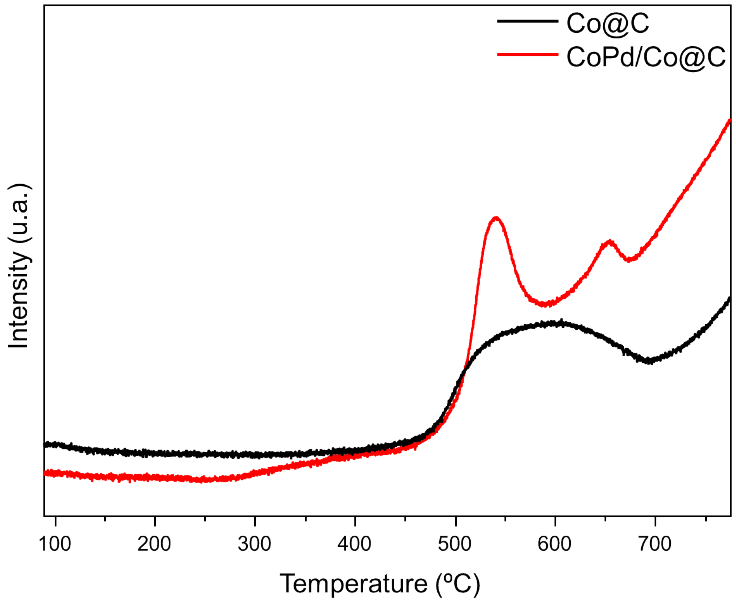

Figure S10.4. CO-TPD analysis for **Co@C** (black) and **CoPd/Co@C** (red).

S11. MIH operating in intermittent conditions

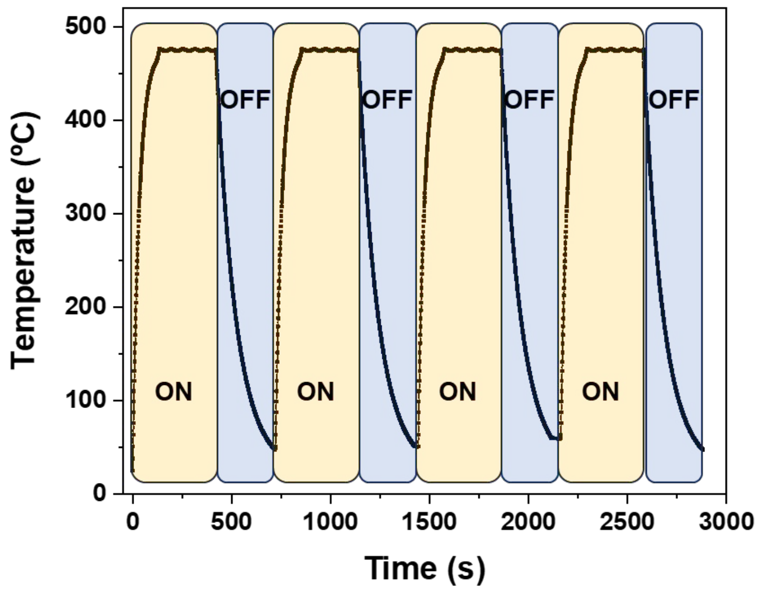

Figure S11.1. CoPd/Co@C heated by MIH, demonstrating its ability to operate under intermittent conditions.

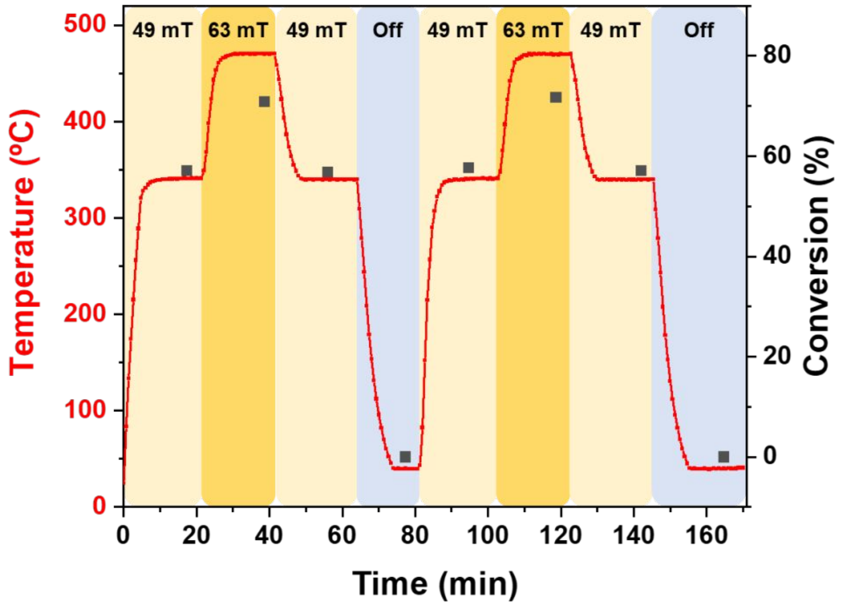

Figure S11.2. CoPd/Co@C heated by MIH, highlighting its capacity to rapidly reach the reaction temperature. CO_2 conversions at different magnetic field applied (49 and 63 mT) are included to confirm the reproducibility of the system.

S12. VSM

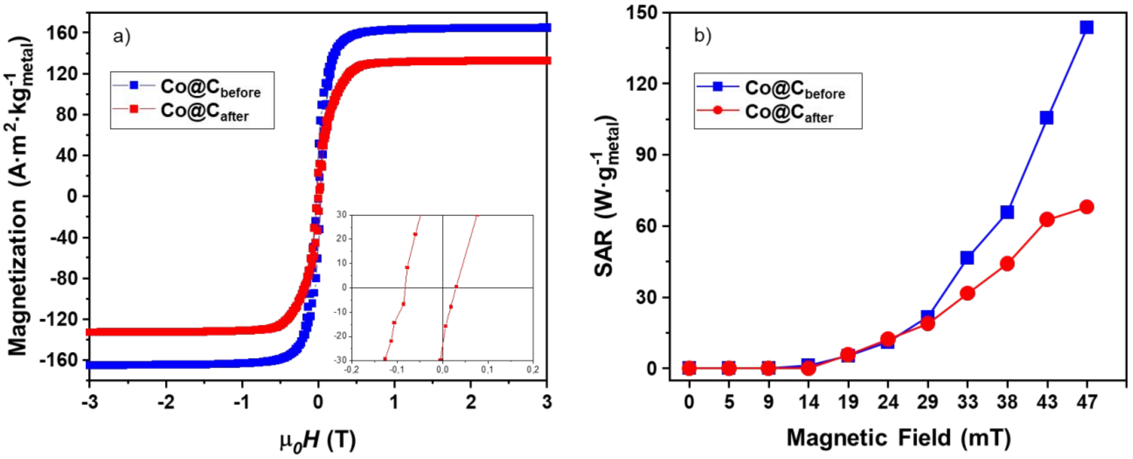

Figure S12.1. Magnetic properties of **Co@C**. Hysteresis loops measured by VSM (a) and SAR measurements (b) of **Co@C** before (blue) and after (red) catalysis.

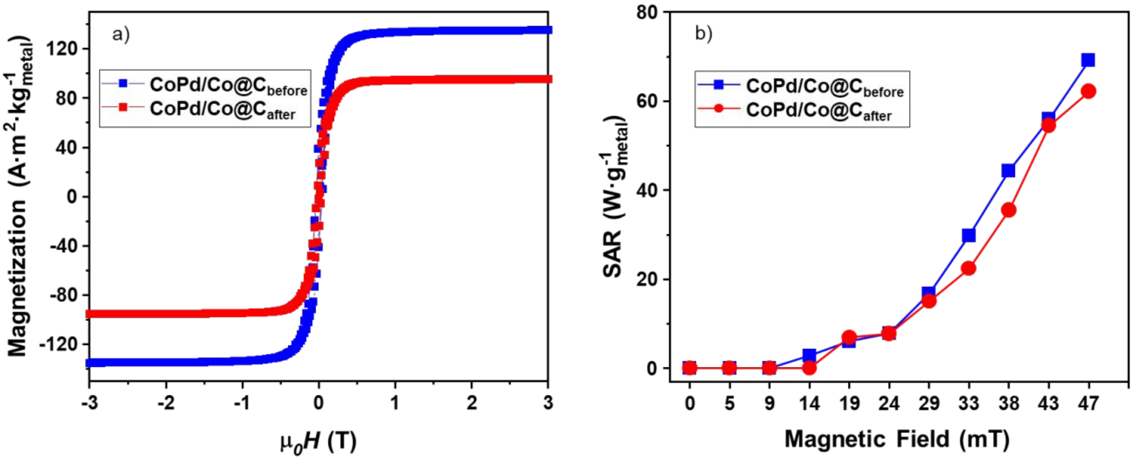

Figure S12.2. Magnetic properties of **CoPd/Co@C**. Hysteresis loops were measured by VSM (a) and SAR measurements of **CoPd/Co@C** before (blue) and after (red) catalysis.

S13. HRTEM and STEM images

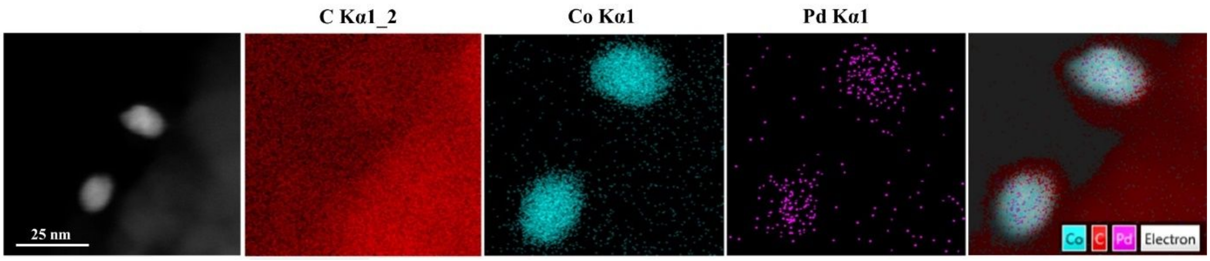

Figure S13.1. STEM-HAADF coupled with EDX analysis of **CoPd/Co@C**, where Co is marked in blue, Pd is marked in purple and C in red.

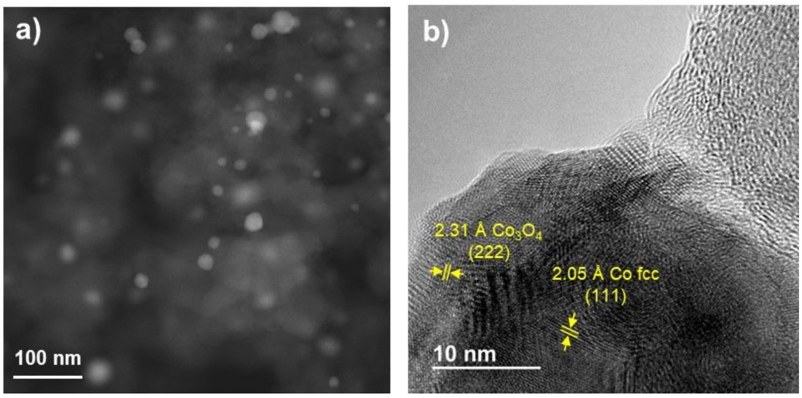

Figure S13.2. STEM image of **Co@C** after catalysis(a), and HRTEM micrographs of **Co@C** after catalysis (b). Lattice spacings are highlighted in yellow.

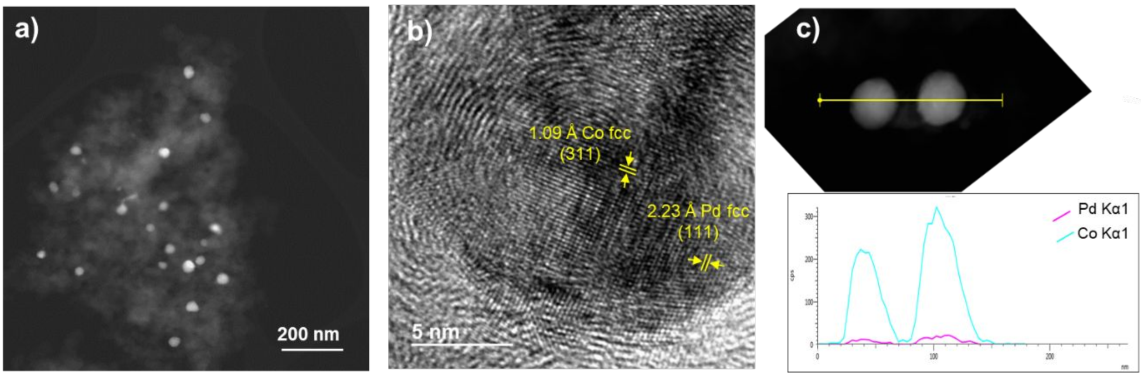

Figure S13.3. a) STEM image of **CoPd/Co@C** after catalysis, b) HRTEM micrographs of **CoPd/Co@C** after catalysis. Lattice spacings are highlighted in yellow, c) STEM- HADDF image and EDX line scan profile of **CoPd/Co@C** after catalysis.

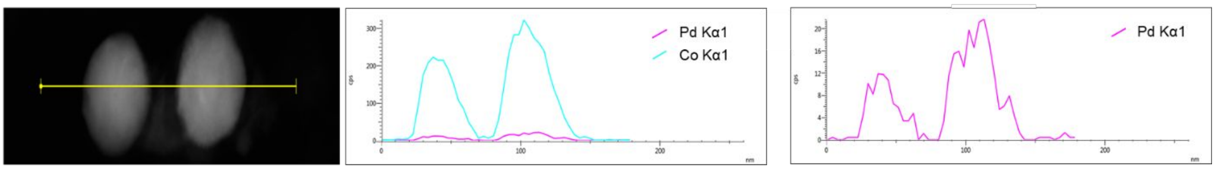

Figure S13.4. EDX line-scan of **CoPd/Co@C** after catalysis.

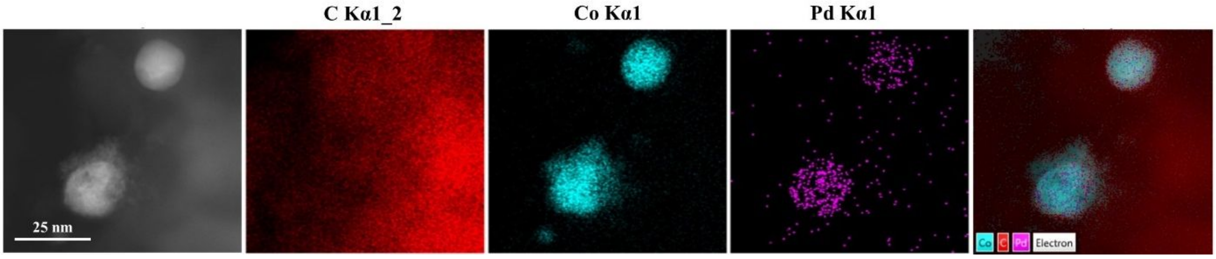

Figure S13.5. STEM-HAADF coupled with EDX analysis of **CoPd/Co@C** after catalysis, where Co is marked in blue, Pd is marked in purple and C in red.

References

(1) Stobinski, L.; Lesiak, B.; Malolepszy, A.; Mazurkiewicz, M.; Mierzwa, B.; Zemek, J.; Jiricek, P.; Bieloshapka, I. Graphene Oxide and Reduced Graphene Oxide Studied by the XRD, TEM and Electron Spectroscopy Methods. *J. Electron Spectros. Relat. Phenomena* **2014**, *195*, 145–154.

(2) Hoekstra, J.; Beale, A. M.; Soulimani, F.; Versluijs-Helder, M.; Geus, J. W.; Jenneskens, L. W. Base Metal Catalyzed Graphitization of Cellulose: A Combined Raman Spectroscopy, Temperature-Dependent X-Ray Diffraction and High-Resolution Transmission Electron Microscopy Study. *J. Phys. Chem. C* **2015**, *119*, 10653–10661.

(3) Zhou, X.; Price, G. A.; Sunley, G. J.; Copéret, C. Small Cobalt Nanoparticles Favor Reverse Water-Gas Shift Reaction Over Methanation Under CO_2 Hydrogenation Conditions. *Angew. Chemie Int. Ed.* **2023**, *62*, e202314274.

(4) Wang, M.; Zhang, G.; Zhu, J.; Li, W.; Wang, J.; Bian, K.; Liu, Y.; Ding, F.; Song, C.; Guo, X. Unraveling the Tunable Selectivity on Cobalt Oxide and Metallic Cobalt Sites for CO_2 Hydrogenation. *Chem. Eng. J.* **2022**, *446*, 137217.

(5) Lu, X.; Liu, Y.; He, Y.; Kuhn, A. N.; Shih, P. C.; Sun, C. J.; Wen, X.; Shi, C.; Yang, H. Cobalt-Based Nonprecious Metal Catalysts Derived from Metal-Organic Frameworks for High-Rate Hydrogenation of Carbon Dioxide. *ACS Appl. Mater. Interfaces* **2019**, *11*, 27717–27726.

(6) Dai, B.; Zhou, G.; Ge, S.; Xie, H.; Jiao, Z.; Zhang, G.; Xiong, K. CO_2 Reverse Water-Gas Shift Reaction on Mesoporous M-CeO_2 Catalysts. *Can. J. Chem. Eng.* **2017**, *95*, 634–642.

(7) Li, Y.; Zhao, Z.; Lu, W.; Zhu, H.; Sun, F.; Mei, B.; Jiang, Z.; Lyu, Y.; Chen, X.; Guo, L.; Wu, T.; Ma, X.; Meng, Y.; Ding, Y. Single-Atom Co-N-C Catalysts for High-Efficiency Reverse Water-Gas Shift Reaction. *Appl. Catal. B Environ.* **2023**, *324*, 122298.

(8) Osakoo, N.; Tawachkultanadilok, P.; Loiha, S.; Roessner, F.; Poo-arporn, Y.; Kidkhunthod, P.; Chanlek, N.; Prayoonpokarach, S.; Wittayakun, J. Green Reduction Route via Ethanol Dehydrogenation and Decomposition for Pd-Promoted Co_3O_4/SBA-15 Catalysts in Reverse Water Gas Shift Reaction: An Operando Time-Resolved X-Ray Absorption Spectroscopy

Investigation. *Appl. Catal. B Environ.* **2022**, *316*, 121670.

(9) Chernyak, S. A.; Suslova, E. V.; Egorov, A. V.; Maslakov, K. I.; Savilov, S. V.; Lunin, V. V. Effect of Co Crystallinity on Co/CNT Catalytic Activity in CO/CO_2 Hydrogenation and CO Disproportionation. *Appl. Surf. Sci.* **2016**, *372*, 100–107.

(10) Iloy, R. A.; Jalama, K.; Khangale, P. R. Effect of a Second Promoter on the Performance of a Potassium Doped Silica-Supported Cobalt Catalyst During CO_2 Hydrogenation to Hydrocarbons. *Catal. Letters* **2024**, *154*, 2818–2828.

(11) Kwak, J. H.; Kovarik, L.; Szanyi, J. Heterogeneous Catalysis on Atomically Dispersed Supported Metals: CO_2 Reduction on Multifunctional Pd Catalysts. *ACS Catal.* **2013**, *3*, 2094–2100.

(12) Cerezo-Navarrete, C.; Marin, I. M.; Marini, C.; Chaudret, B.; Martínez-Prieto, L. M. Structural Transformation of Carbon-Encapsulated Core-Shell CoNi Nanoparticles during Magnetically Induced CO_2 Reduction into CO. *Appl. Catal. B Environ. Energy* **2024**, *347*, 123780.

(13) Chen, J.; Su, S.; Wang, C.; Li, Q.; Wang, H.; Xu, W.; Li, X.; Jia, H. Understanding CO_2 Reduction via Reverse Water-Gas Shift Triggered by Electromagnetic Induction at Moderate Condition. *Chem. Eng. J.* **2023**, *476*, 146712.
